# Supplementary material for: Enantiocontrolled Preparation of ϒ-Substituted Cyclohexenones: Synthesis and Kinase Activity Assays of Cyclopropyl-Fused Cyclohexane Nucleosides
Source: Int J Mol Sci. 2022 Aug 26;23(17):9704. doi: 10.3390/ijms23179704 (PMC9456008; doi:10.3390/ijms23179704)
Supplement: Supplementary file 1 [file ijms-23-09704-s001.zip › ijms-1853459-supplementary.pdf]

Supplementary Information for:

Enantiocontrolled preparation of ϒ-substituted cyclohexenones: synthesis and kinase activity assays of cyclopropyl-fused cyclohexane nucleosides

Sergio Jurado, [1] Beatriz Domínguez-Pérez,[1] Ona Illa, [1] Jan Balzarini, [2] Félix Busqué [1,*] and Ramon Alibés [1,*]

[1] Departament de Química, Universitat Autònoma de Barcelona, Bellaterra 08193, Barcelona, Spain.

[2] Laboratory of Virology and Chemotherapy (Rega Institute). KU Leuven, B-3000 Leuven, Belgium.

ramon.alibes@uab.cat; felix.busque@uab.cat

Table of contents

Asymmetric Transfer Hydrogenation (ATH) study on other cycloalkenones of five and seven-membered rings (Table S1)	S4
Preparation of 1,4-dioxaspiro[4.5]dec-6-en-8-one, **4**	S6
Preparation of (4*R*)-4-hydroxycyclopent-2-en-1-one, (*R*)-**S5**	S6
Obtention of cyclopentanone, **S7**, and cyclopentan-1-ol, **S8**	S7
Preparation of (4*R*)-4-Acetoxycyclohept-2-en-1-one, (*R*)-**S11**	S8
Preparation of (1*R*)-2-cyclohepten-1-ol, (*R*)-**S12**	S9
^{1}H- and ^{13}C-NMR spectra of compound **4**	S10
^{1}H- and ^{13}C-NMR spectra of compound (*R*)-**6**	S11
^{1}H-NMR spectrum of compound (*S*)-**6**	S12
^{1}H- and ^{13}C-NMR spectra of compound (*R*)-**1a**	S13
^{1}H-NMR spectrum of compound (*S*)-**1a**	S14
^{1}H-NMR spectrum of compound (*R*)-**1b**	S15
^{1}H-NMR spectrum of compound (*S*)-**1b**	S15
^{1}H-NMR spectrum of mixture of compounds (*R*)-**11** and **12**	S16
^{1}H-NMR spectrum of compound (*R*)-**13**	S16
^{1}H-NMR spectrum of compound (*R*)-**15**	S17
^{1}H- and ^{13}C-NMR spectra of compound **16**	S18
NOESY and COSY experiments for compound **16**	S19
HSQC experiment for compound **16**	S20
^{1}H- and ^{13}C-NMR spectra of compound **18**	S21
^{1}H- and ^{13}C-NMR spectra of compound **19**	S22
^{1}H- and ^{13}C-NMR spectra of compound **20**	S23
^{1}H- and ^{13}C-NMR spectra of a mixture of compounds **21** and **22**	S24
NOESY experiment for a mixture of compounds **21** and **22**	S25
^{1}H- and ^{13}C-NMR spectra of compound **23**	S26
^{1}H- and ^{13}C-NMR spectra of compound **24**	S27
^{1}H- and ^{13}C-NMR spectra of compound **25**	S28
^{1}H- and ^{13}C-NMR spectra of compound **27**	S29
^{1}H- and ^{13}C-NMR spectra of compound **2e**	S30
^{1}H-NMR spectrum of compounds (*R*)-**S5** and **S6**	S31
^{1}H-NMR spectrum of compounds **S2**, **S7** and **S8**	S31
^{1}H-NMR spectrum of compound (*R*)-**S11**	S32
^{1}H-NMR spectrum of compound (*R*)-**S12**	S32
Enantiomeric purity	S33

CHPLC of compounds (*R*)- and (*S*)-**1a** S33

CHPLC of compounds (*R*)- and (*S*)-**11** S34

CHPLC of compounds (*R*)- and (*S*)-**13** S35

CHPLC of compounds (*R*)- and (*S*)-**15** S36

CHPLC of compounds (*R*)- and (*S*)-**S5** S37

CHPLC of compounds (*R*)- and (*S*)-**S11** S38

CHPLC of compounds (*R*)- and (*S*)-**S12** S39

Antiviral Activity Assays. Methods S40

Cytotoxicity and antiherpetic activity (Table S2) S40

References S41

Asymmetric Transfer Hydrogenation (ATH) study on other cycloalkenones of five and seven-membered rings.

The ATH reaction has also been studied with the cycloalkenones **S1-S4** of five- and seven-membered rings using the optimized conditions ((*R*,*R*)-5, TBAC, and HCOONa in CH_2Cl_2/H_2O 1:1, Table S1).

Table S1. Asymmetric Hydrogenation of Cycloalkenones **S1-S4**.

Entry	Substrate	Products	Yield (%)	Ratio[a]	%ee[b]
**1**	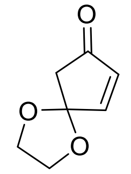 **S1**	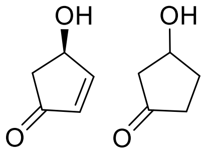 **S5** **S6**	41	2:1	20
**2**	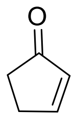 **S2**	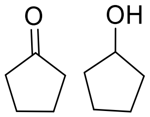 **S7** **S8**	n.a.[c]	1:1	--
**3**	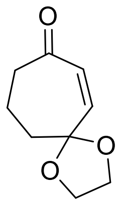 **S3**	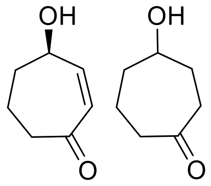 **S9** **S10**	60	3:1	70[d]
**4**	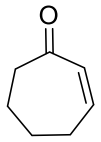 **S4**	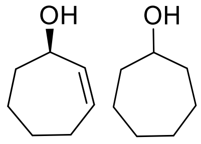 **S12** **S13**	41	7:1	83

[a] Determined by ^{1}H-NMR. [b] Determined by chiral high-pressure liquid chromatography (CHPLC). [c] n.a.: not available. [d] Determined by chiral high-pressure liquid chromatography (CHPLC) on its *O*-acetyl derivative **S11**.

The ATH reduction of 1,4-cyclopentenedione monoethylene ketal **S1** and subsequent hydrolysis of the ketal furnished alcohol **S5** with low enantiomeric excess (20% ee) and its completely hydrogenated derivative **S6** in a 2:1 ratio (entry 1). The asymmetric reduction of the 2-cyclopentenone **S2**, did not afford the expected allylic alcohol but the cyclopentanone **S7** and the corresponding cyclopentanol **S8** (entry 2), suggesting a higher C=C selectivity. The results

suggest that a five-membered ring is detrimental to the enantioselectivity as well as the chemoselectivity of the reaction. The ATH reduction on the 1,4-cycloheptenedione monoethylene ketal **S3** followed by deprotection of the carbonyl group provided the corresponding alcohol **S9** with moderate enantiomeric excess (70% ee) along with the hydrogenated **S10** in a 3:1 ratio (entry 3). Finally, the asymmetric reduction of 2-cycloheptenone **S4** led to allylic alcohol **S12** with better enantioselectivity (83% ee) along with its totally hydrogenated derivative **S13** in a 7:1 ratio (entry 4). Thus, these results also suggest that the enantio- and chemoselectivity of the reaction are dependent on the size of the ring.

Overall, this study showed that among the cycloalkenones investigated, the best results were afforded with six-membered rings in both enantio- and C=O/C=C selectivity.

1,4-Dioxaspiro[4.5]decan-6-en-8-one, 4

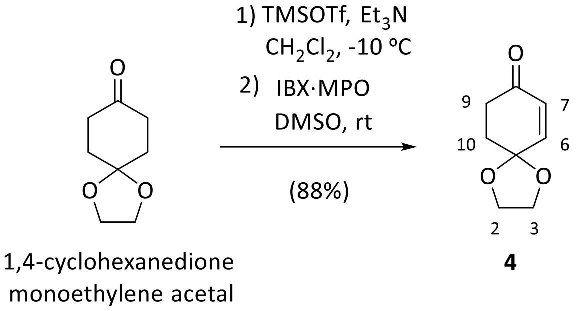

To a stirred solution of 1,4-cyclohexanedione monoethylene acetal (3.00 g, 18.7 mmol) and Et_3N (5.20 mL, 37.3 mmol) in dry CH_2Cl_2 (130 mL) at -10 °C, under nitrogen atmosphere, TMSOTf (4.20 mL, 23.2 mmol) was added dropwise, and the reaction mixture was stirred for 2 h and then concentrated under reduced pressure. Then, a solution of the resulting crude in DMSO (50 mL) was poured onto a solution of IBX (10.4 g, 37.3 mmol) and MPO (4.82 mg, 37.3 mmol) in DMSO (35 mL), that had been previously stirred during 1 h until becoming a clear solution. After 2 h of stirring at rt, CH_2Cl_2 (150 mL) and a saturated aqueous $NaHCO_3$ solution (150 mL) were added. The two phases were separated, and the aqueous layer was extracted with CH_2Cl_2 (2 x 100 mL). The combined organic extracts were concentrated under reduced pressure and washed with a solution of HCl 5% (100 mL) and brine (2 x 100 mL), dried (Na_2SO_4) and concentrated under vacuum to provide the known enone **4** (2.52 g, 16.4 mmol, 88% yield) as a yellow oil: ^1H NMR (250 MHz, $CDCl_3$) δ 6.60 (d, $J_{6,7}$=10.2 Hz, 1H, H-6), 5.99 (d, $J_{7,6}$=10.2 Hz, 1H, H-7), 4.11 – 3.98 (m, 4H, H-2, H-3), 2.62 (t, $J_{9,10}$=6.5 Hz, 2H, H-9), 2.19 (t, $J_{10,9}$=6.5 Hz, 2H, H-10); ^{13}C NMR (90 MHz, $CDCl_3$) δ 198.7 (C=O), 146.4 (C-6), 130.6 (C-7), 104.0 (C-5), 65.1 (C-2, C-3), 35.3/32.9 (C-9, C-10).

(4*R*)-4-hydroxycyclopent-2-en-1-one, (*R*)-S5

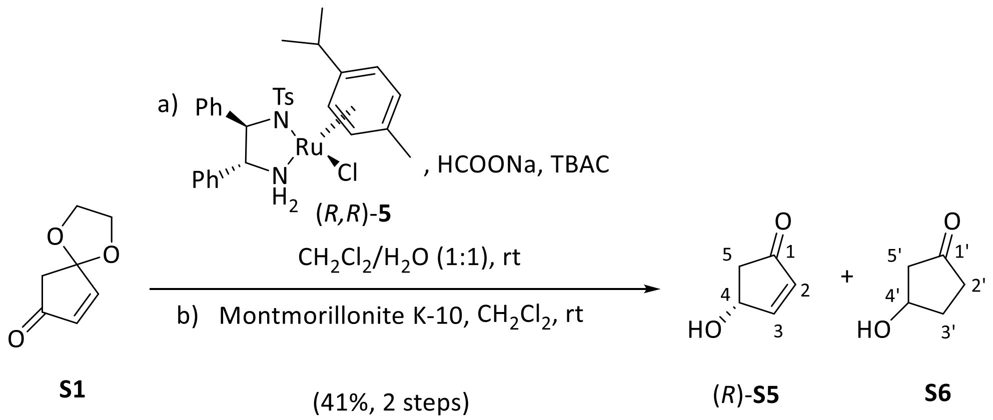

To a stirred solution of enone **S1** (28 mg, 0.20 mmol) in a biphasic media of CH_2Cl_2 (0.7 mL) and water (0.7 mL) and under a nitrogen flux, tetrabutylammonium chloride (19 mg, 0.07 mmol),

sodium formate (46.3 mg, 0.68 mmol) and (*R,R*)-**5** (4.3 mg, 0.007 mmol) were sequentially added at rt. The reaction mixture was allowed to stir for 5 h. Then, CH_2Cl_2 (1 mL) and water (1 mL) were added, the two phases were separated, and the aqueous layer was extracted with CH_2Cl_2 (3 x 0.5 mL). The volatiles were removed under reduced pressure and the resulting crude was dissolved in CH_2Cl_2 (1.9 mL) and Montmorillonite K-10 (105 mg) was added. The mixture was stirred at rt for 2 h. Then, it was filtered, and the solvent removed under vacuum to provide a 2:1 mixture of enone (*R*)-**S5** and keto alcohol **S6** (8 mg, 0.08 mmol, 41% yield for the two steps) as a pale-yellow oil. CHPLC (Daicel Chiralcel IC): 20% ee; ^{1}H NMR (250 MHz, $CDCl_3$) ((*R*)-**S5**) δ 7.56 (dd, $J_{3,2}$=5.7 Hz, $J_{3,4}$=2.4 Hz, 1H, H-3), 6.24 (dd, $J_{2,3}$= 5.7 Hz, $J_{2,4}$=1.3 Hz, 1H, H-2), 5.06 (br s, 1H, H-4), 2.79 (dd, J_{gem}=18.5 Hz, $J_{5,4}$=6.1 Hz, 1H, H-5), 2.28 (dd, J_{gem}=18.5 Hz, $J_{5,4}$=2.2 Hz, 1H, H-5); (**S6**) δ: 4.64 (s, 1H, H-4'), 2.59 – 2.37 (m, 2H, H-5), 2.31 – 2.02 (m, 4H, H-2, H-3).

Cyclopentanone, S7, and cyclopentan-1-ol, S8

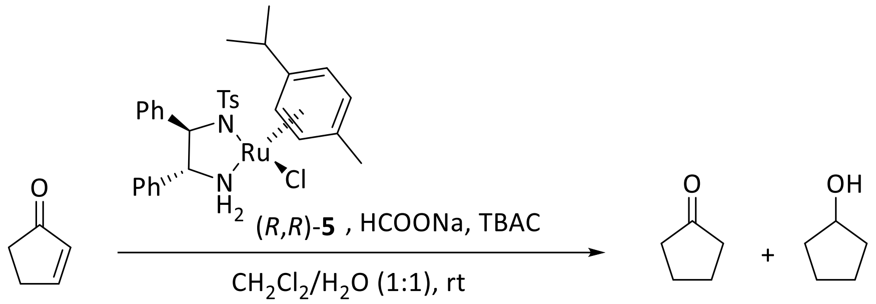

S2 **S7** **S8**

To a stirred solution of enone **S2** (500 µL, 5.85 mmol) in a biphasic media of CH_2Cl_2 (19 mL) and water (19 mL) and under a nitrogen flux, tetrabutylammonium chloride (493 mg, 1.79 mmol), sodium formate (597 mg, 8.77 mmol) and (*R,R*)-**5** (110 mg, 0.18 mmol) were sequentially added at rt. The reaction mixture was allowed to stir for 24 h. After that time, more catalyst (*R,R*)-**5** (110 mg, 0.18 mmol) was added and the reaction was stirred for further 24 h. Then, a third batch of catalyst (*R,R*)-**5** (110 mg, 0.18 mmol) was added and the mixture was stirred for 24 h. Then, CH_2Cl_2 (20 mL) and water (20 mL) were added, the two phases were separated, and the aqueous layer was extracted with CH_2Cl_2 (3 x 20 mL). The volatiles of the organic phase were removed under reduced pressure to furnish a mixture of **S7**, **S8**, and starting material **S2**, in a 3:3:2 ratio as a yellowish oil. ^{1}H NMR (250 MHz, $CDCl_3$) (**S2**) δ 7.75 (dt, $J_{3,2}$=5.4 Hz, $J_{3,4}$=$J_{3,4}$=2.7 Hz, 1H, H-3), 6.23 (dt, $J_{2,3}$=5.4 Hz, $J_{2,5}$=$J_{2,5}$=2.3 Hz, 1H, H-2), 2.72 (dq, $J_{5,4}$=7.0 Hz, $J_{5,2}$=$J_{5,3}$=2.3 Hz, 2H, H-5), 2.41 – 2.35 (m, 2H, H-4); (**S7**) δ: 2.25 – 2.11 (m, 4H, H-2, H-5), 2.05 – 1.90 (m, 4H, H-3, H-4); (**S8**) δ: 4.48 – 4.26 (m, 1H, H-1), 3.38 (s, 1H, -OH), 1.85 – 1.72 (m, 4H, H-2, H-5), 1.66 – 1.54 (m, 4H, H-3, H-4).

(4*R*)-4-Acetoxycyclohept-2-en-1-one, (*R*)-S11

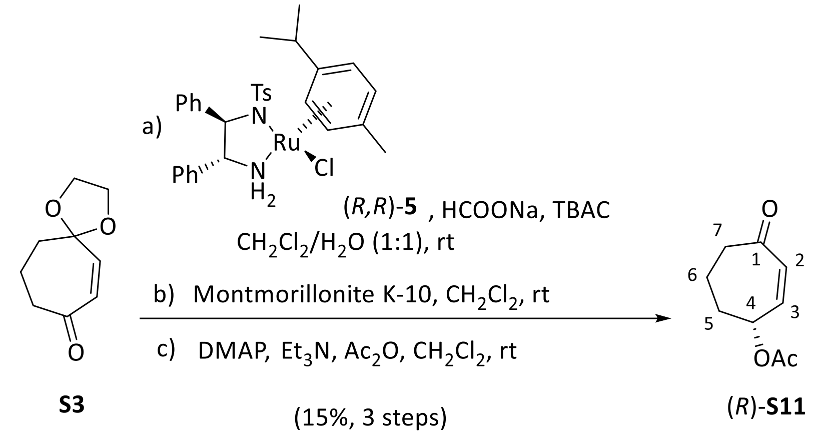

To a stirred solution of enone **S3** (35 mg, 0.21 mmol) in a biphasic media of CH_2Cl_2 (0.69 mL) and water (0.69 mL) and under a nitrogen flux, tetrabutylammonium chloride (17 mg, 0.07 mmol), sodium formate (42 mg, 0.62 mmol) and (*R,R*)-**5** (4 mg, 0.006 mmol) were sequentially added at rt. The reaction mixture was allowed to stir for 5h. Then, CH_2Cl_2 (20 mL) and water (20 mL) were added, the two phases were separated, and the aqueous layer was extracted with CH_2Cl_2 (3 x 10 mL). The volatiles were removed under reduced pressure and the resulting crude was dissolved in CH_2Cl_2 (2.6 mL) and montmorillonite K-10 (177 mg) was added. The mixture was stirred at rt for 2 h. Then, it was filtered, and the solvent removed under vacuum to provide a chromatographically inseparable 3:1 mixture of enone **S9** and keto alcohol **S10** (16 mg, 0.12 mmol, 60% yield for the two steps) as a pale yellow oil.

To a stirred solution of the reaction crude in dry CH_2Cl_2 (0.16 mL), DMAP (0.59 mg, 4.75 µmol) and Et_3N (22 µL, 0.16 mmol) were added followed by acetic anhydride (10 µL, 0.11 mmol) dropwise. After stirring for 10 min, the reaction mixture was diluted with diethyl ether (1 mL) and a saturated aqueous $NaHCO_3$ solution (1 mL). After separation, the aqueous layer was extracted with diethyl ether (3 x 1 mL) and the combined organics were washed with a saturated aqueous $NaHCO_3$ solution and brine. The volatiles of the organic phase were removed under reduced pressure and the resulting residue was purified by column chromatography (hexanes:EtOAc 4:1) to furnish the protected alcohol (*R*)-**S11** (3 mg, 0.02 mmol, 15% yield for the three steps) as a yellowish oil: CHPLC (Daicel Chiralcel AD-H): 70% ee; $[\alpha]_D^{20}$= +90.2 (*c* 0.27, $CHCl_3$) [lit:[1] $[\alpha]_D^{20}$= + 113.2 (*c* 0.5, $CHCl_3$), 86% ee]; ^1H NMR (250 MHz, $CDCl_3$) δ 6.43 (ddd, $J_{3,2}$=12.6 Hz, $J_{3,4}$=3.4 Hz, $J_{3,5}$=1.1 Hz, 1H, H-3), 6.02 (dd, $J_{2,3}$=12.6 Hz, $J_{2,4}$=2.2 Hz, 1H, H-2), 5.67 – 5.51 (m, 1H, H-4), 2.71 – 2.56 (m, 2H, H-7), 2.27 – 2.12 (m, 1H, H-5), 2.10 (s, 3H, -CH_3), 1.96 – 1.81 (m, 3H, H-5, 2H-6).

(1*R*)-2-cyclohepten-1-ol, (*R*)-S12

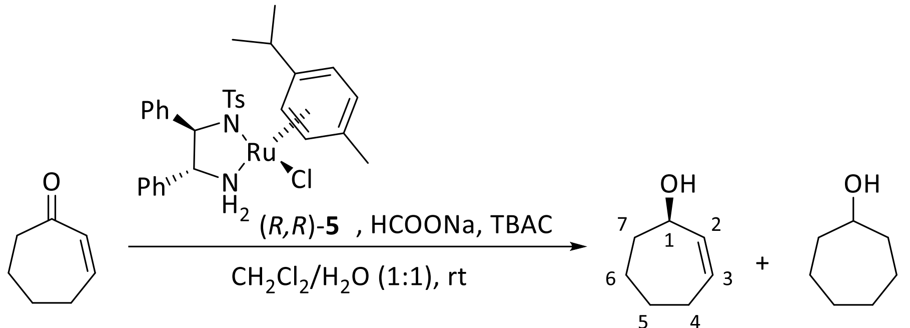

S4 (*R*)-**S12** **S13**

To a stirred solution of enone **S4** (500 µL, 3.59 mmol) in a biphasic media of CH_2Cl_2 (12 mL) and water (12 mL) and under a nitrogen flux, tetrabutylammonium chloride (299 mg, 1.08 mmol), sodium formate (732 mg, 10.8 mmol) and (*R,R*)-**5** (68.5 mg, 0.11 mmol) were sequentially added at rt. The reaction mixture was allowed to stir for 24 h. Then, CH_2Cl_2 (12 mL) and water (12 mL) were added, the two phases were separated, and the aqueous layer was extracted with CH_2Cl_2 (3 x 5 mL). The volatiles of the organic phase were removed under reduced pressure to furnish a 7:1 mixture of allylic alcohol (*R*)-**S12** and the totally hydrogenated derivative **S13** which was purified by column chromatography ($CHCl_3$) to furnish the allylic alcohol (*R*)-**S12** (165 mg, 1.47 mmol, 41% yield) as a yellowish oil: CHPLC (Daicel Chiralcel IC): 83% ee; $[\alpha]_D^{20}$= +19.5 (*c* 1.4, $CHCl_3$) [lit:[2] $[\alpha]_D^{20}$= +28.2 (*c* 1.03, $CHCl_3$), >99% ee]; ^{1}H NMR (400 MHz, $CDCl_3$) δ 5.79 – 5.69 (m, 2H, H-2, H-3), 4.42 – 4.36 (m, 1H, H-1), 2.23 – 2.13 (m, 1H, H-7), 2.08 – 1.97 (m, 1H, H-7), 1.96 – 1.82 (m, 2H, H-4), 1.71 – 1.51 (m, 3H, 2H-6, H-5), 1.41 – 1.30 (m, 1H, H-5); ^{13}C NMR (100 MHz, $CDCl_3$) δ: 138.0 (C-2), 130.3 (C-3), 72.3 (C-1), 36.9 (C-7), 28.8 (C-4), 27.0/26.9 (C-5, C-6).

Chroma-tography (pentane/diethyl ether 7:1) afforded alcohol9b(126 mg, 94%) of >99%ee[GC, γ Lipodexg, t_R (9b) = 13.95 min,tR(ent-9b) = 14.06 min] as a colorless oil: [α]20D = 28.2 (c 1.03, CH_2Cl_2)

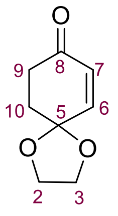

4

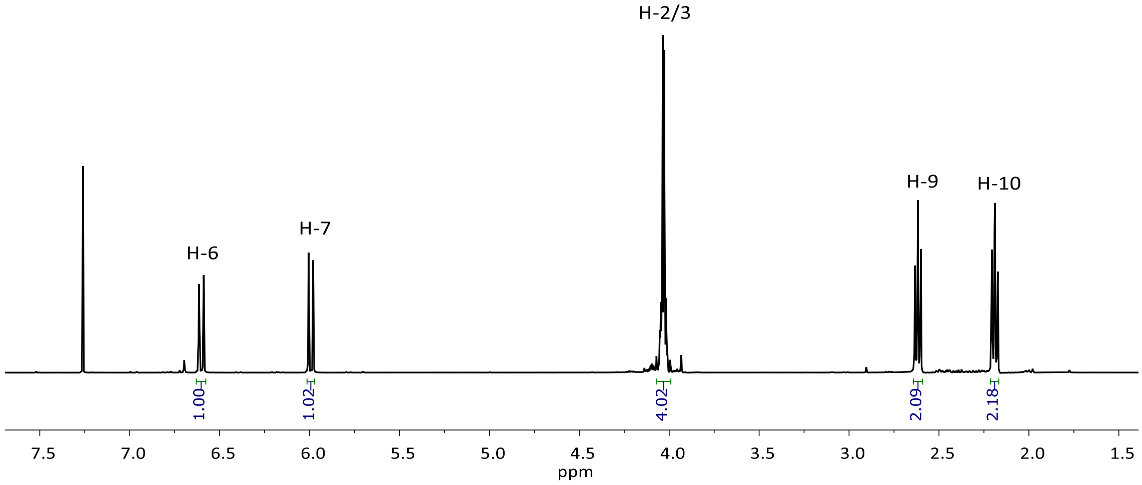

^{1}H NMR (250 MHz, $CDCl_3$)

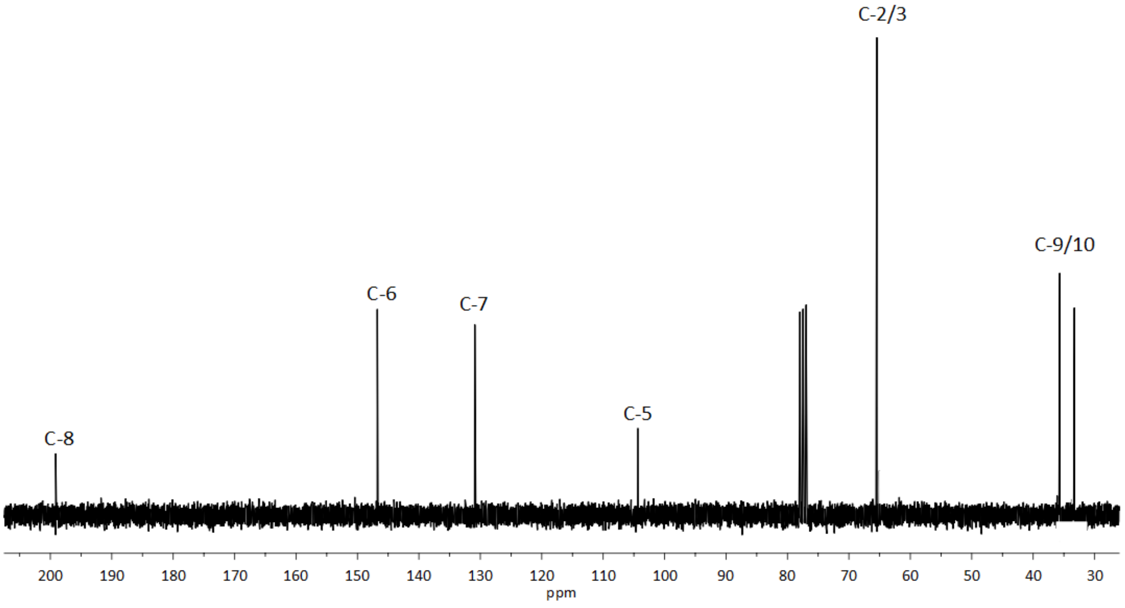

^{13}C NMR (90 MHz, $CDCl_3$)

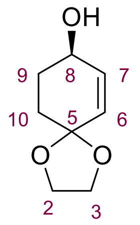

(*R*)-**6**

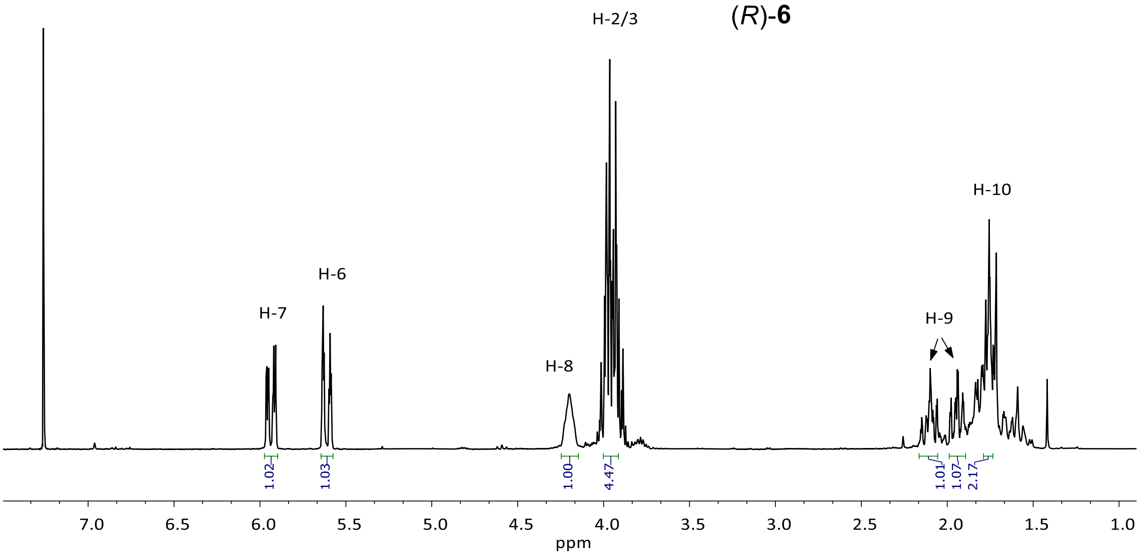

^{1}H NMR (250 MHz, $CDCl_3$)

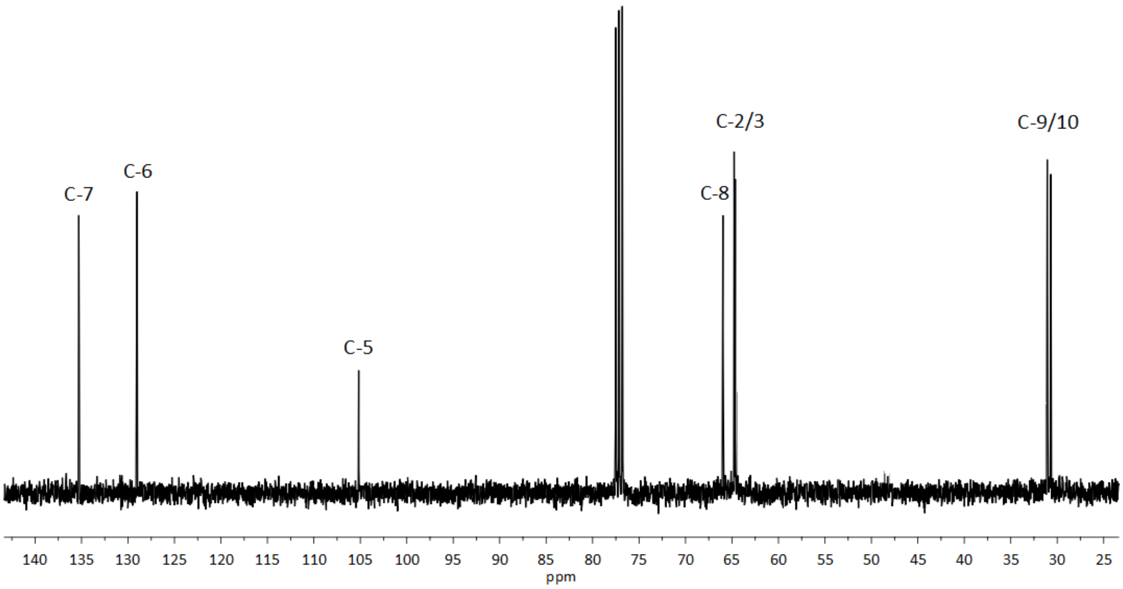

^{13}C NMR (90 MHz, $CDCl_3$)

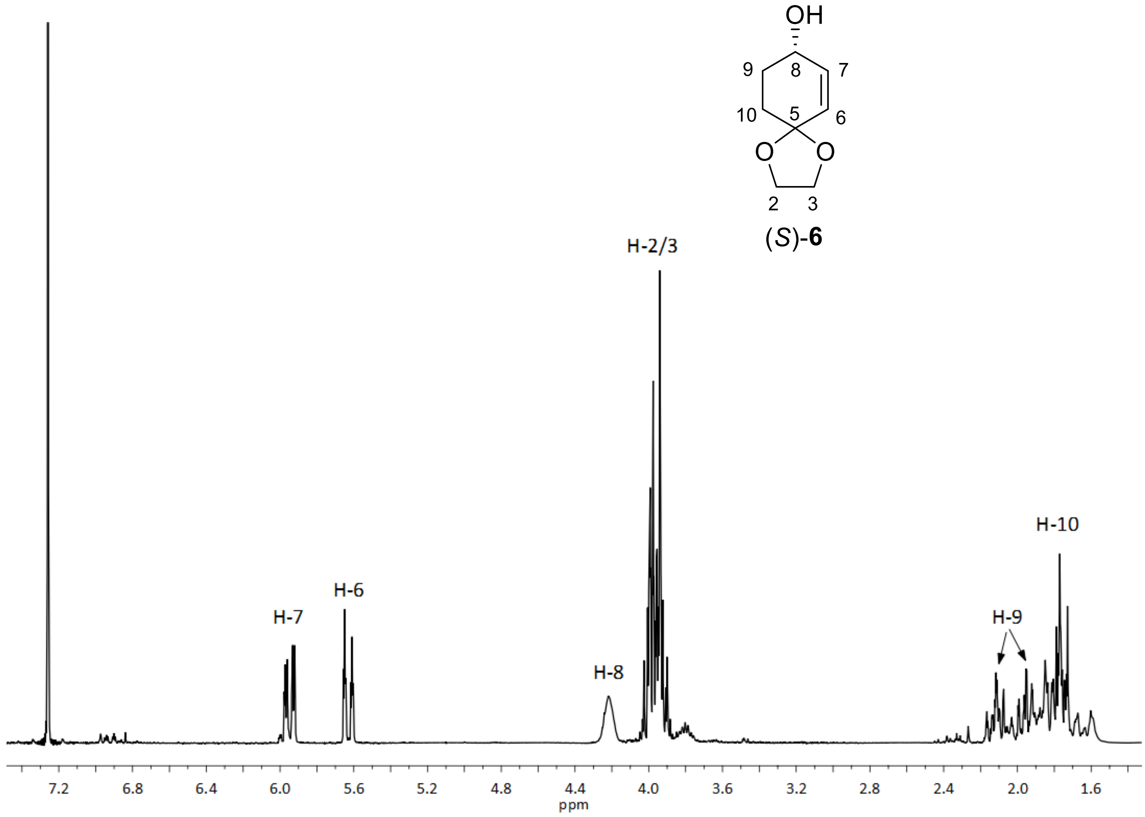

^{1}H NMR (250 MHz, $CDCl_3$)

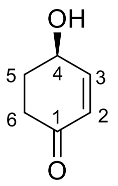

(*R*)-**1a**

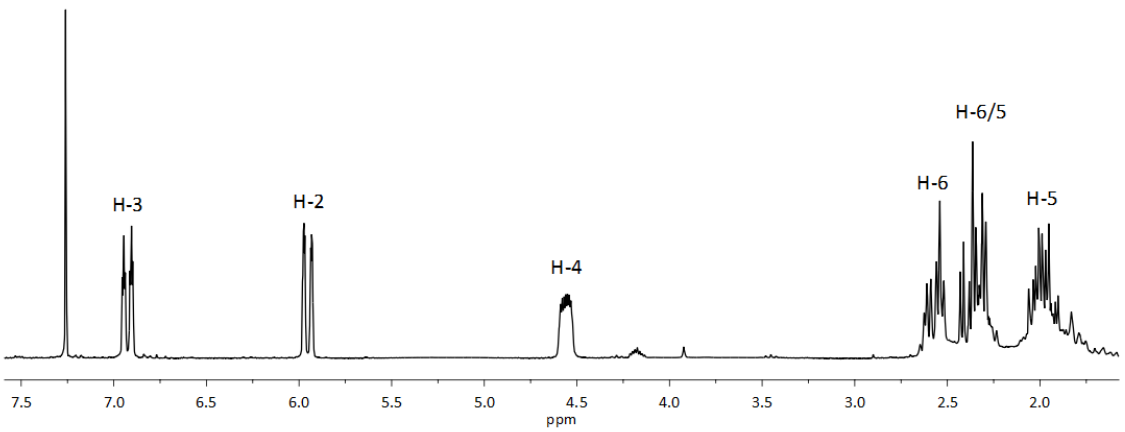

^{1}H NMR (250 MHz, $CDCl_3$)

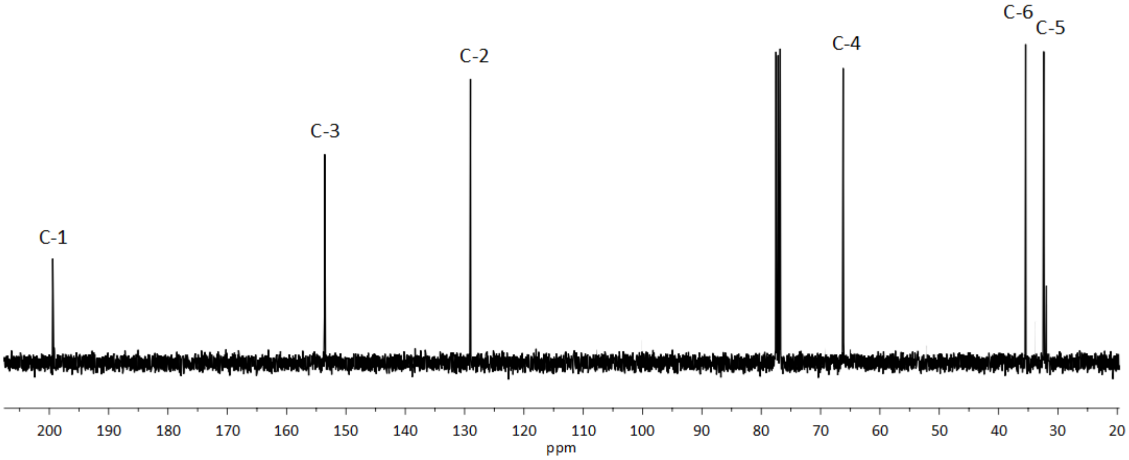

^{13}C NMR (90 MHz, $CDCl_3$)

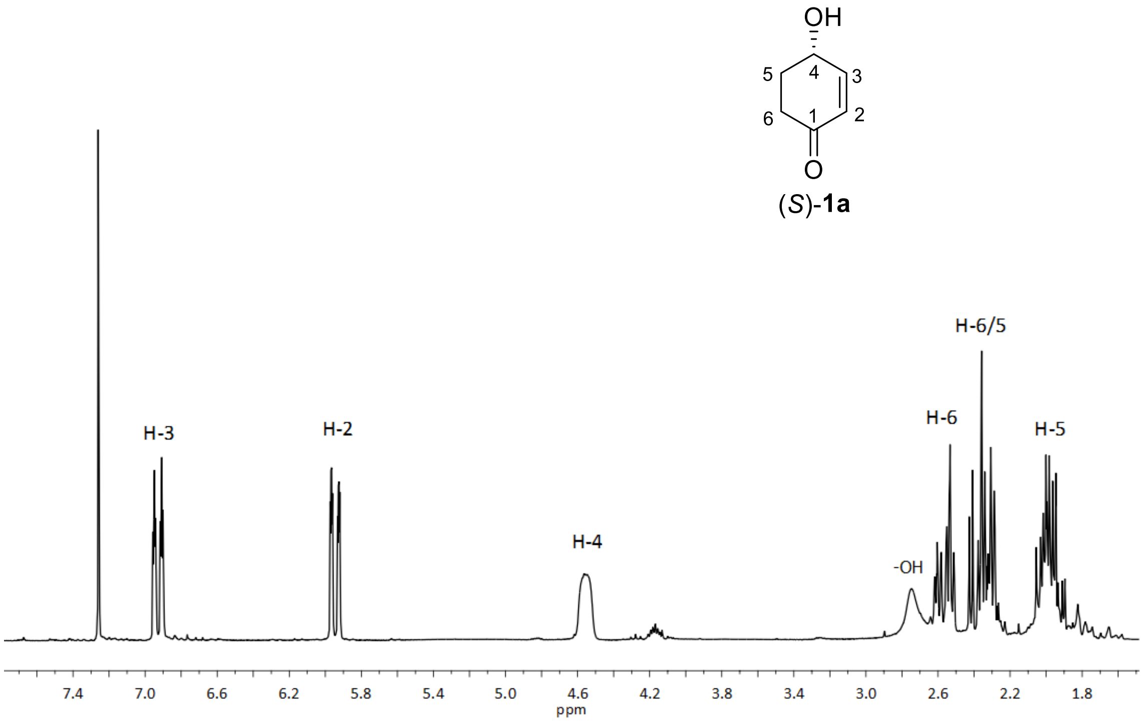

^{1}H NMR (250 MHz, $CDCl_3$)

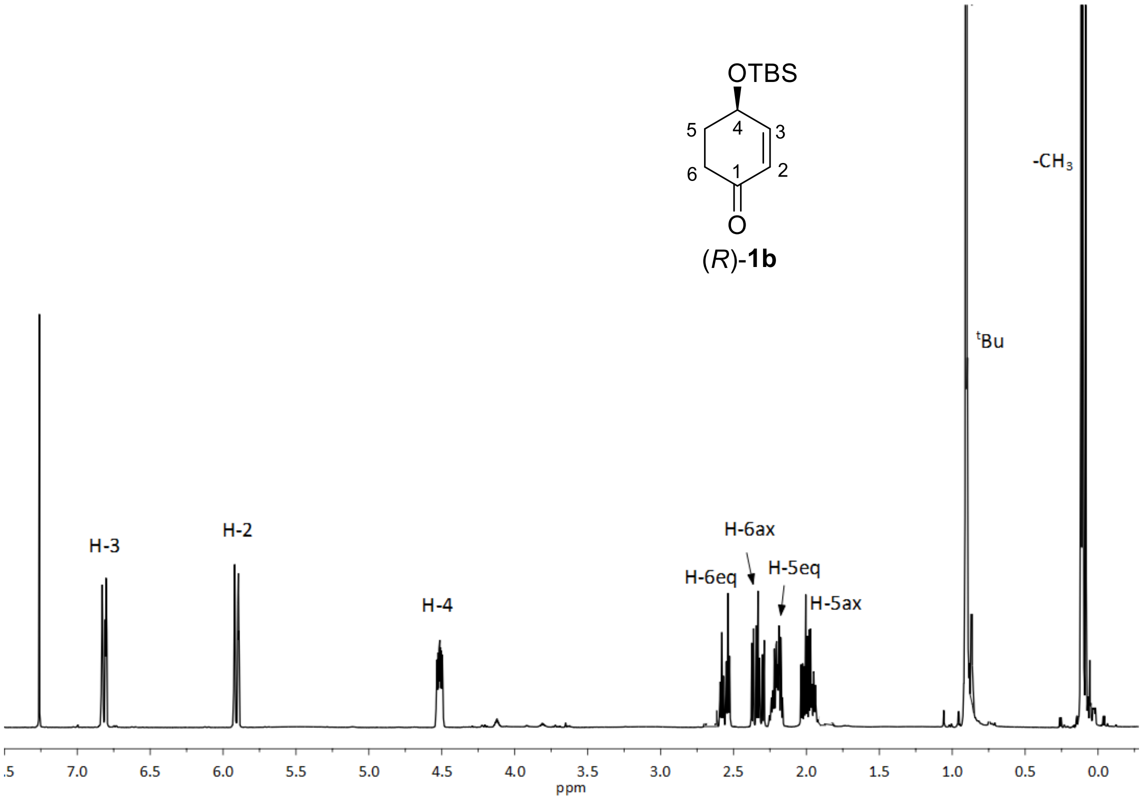

^{1}**H NMR** (400 MHz, $CDCl_3$)

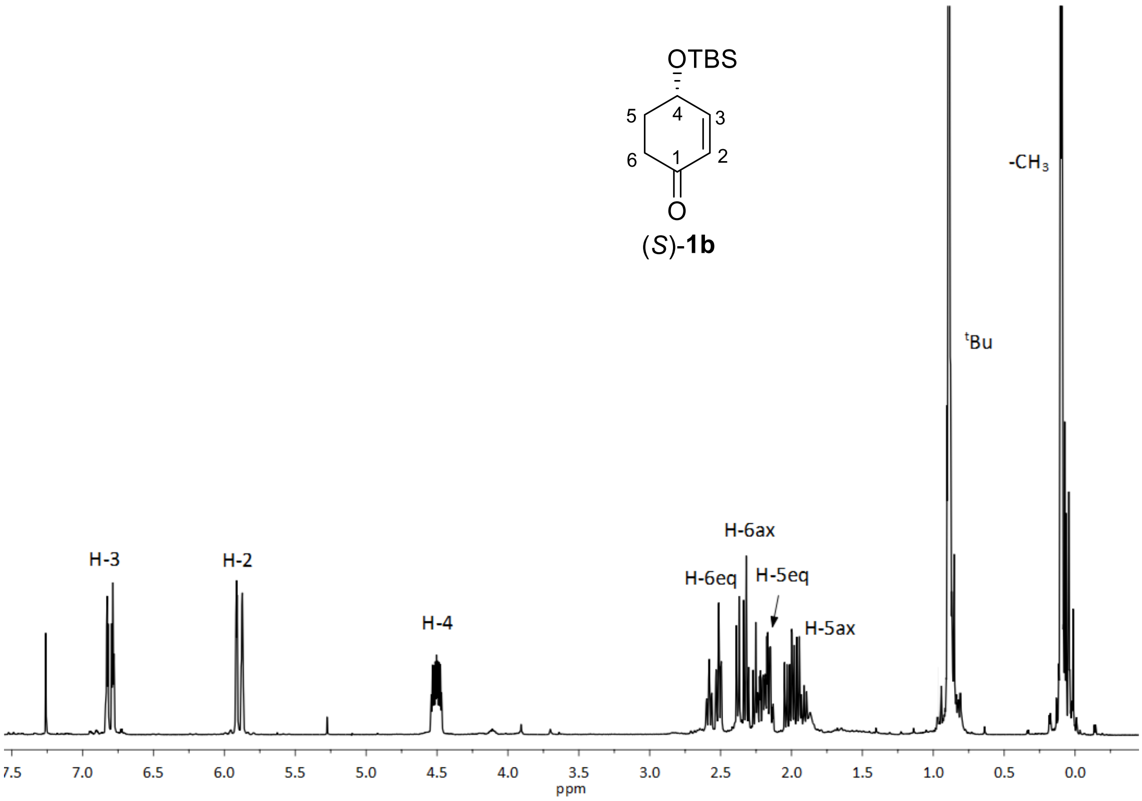

^{1}**H NMR** (400 MHz, $CDCl_3$)

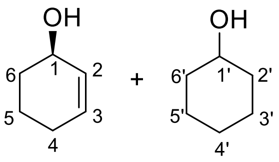

(*R*)-**11** **12**

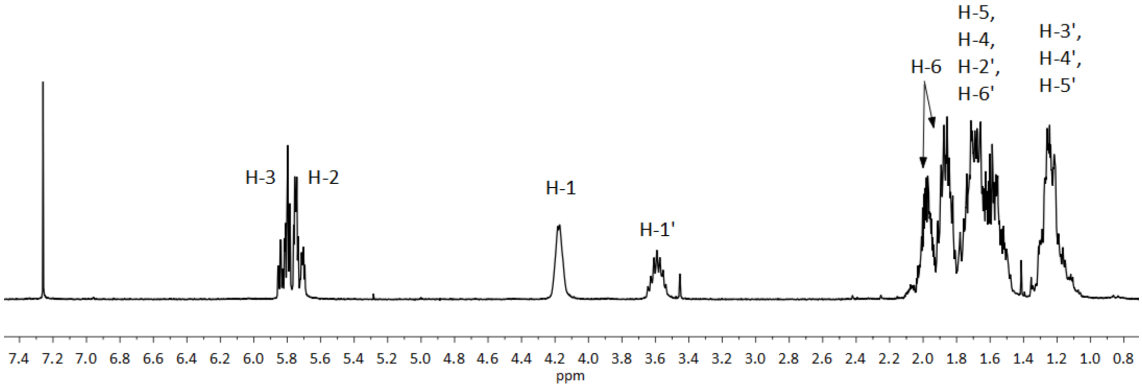

^{1}H NMR (400 MHz, $CDCl_3$)

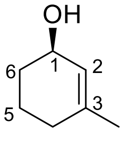

(*R*)-**13**

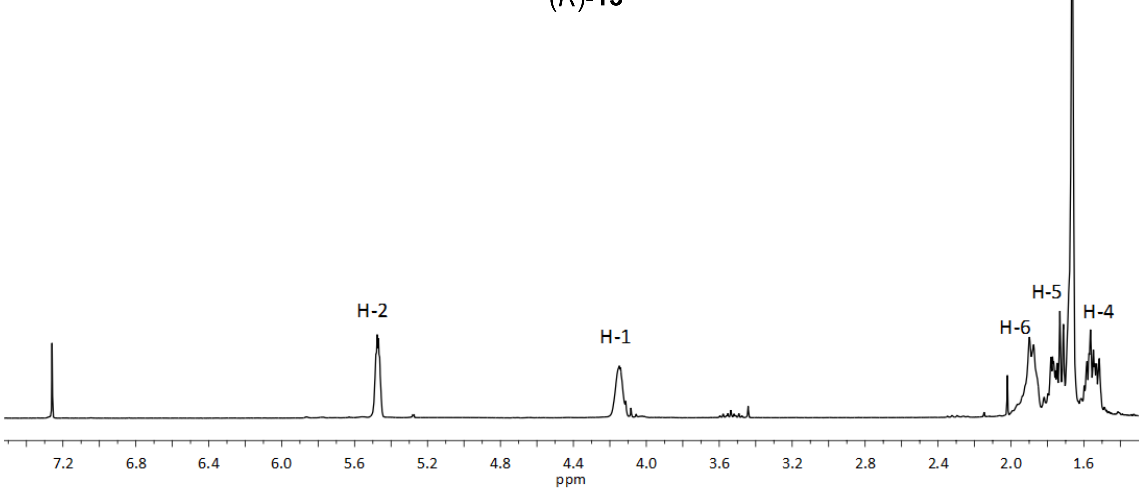

^{1}H NMR (250 MHz, $CDCl_3$)

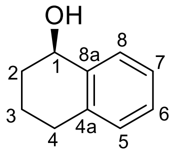

(R)-**15**

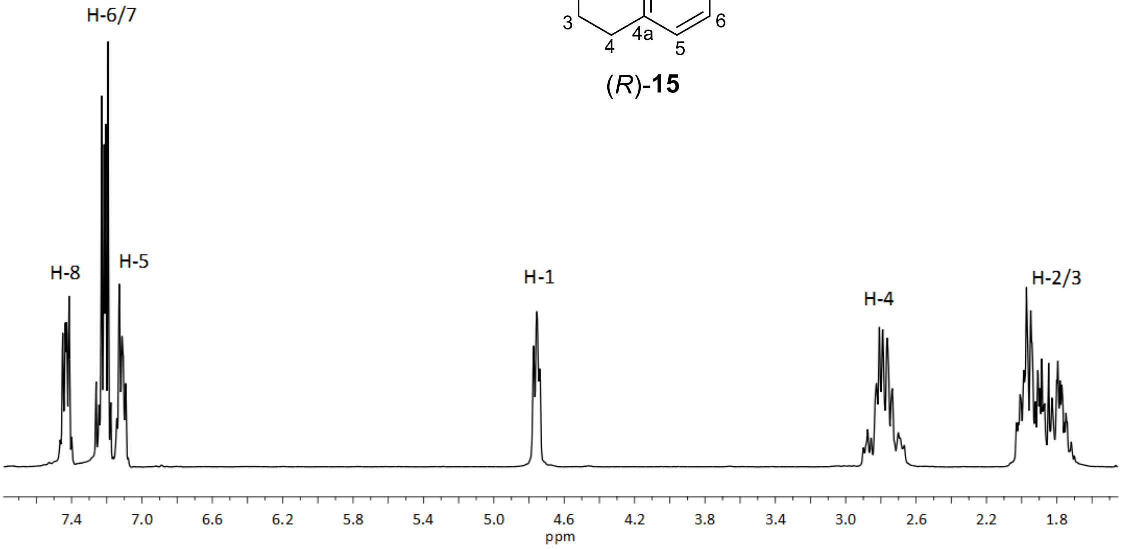

^{1}**H NMR** (250 MHz, $CDCl_3$)

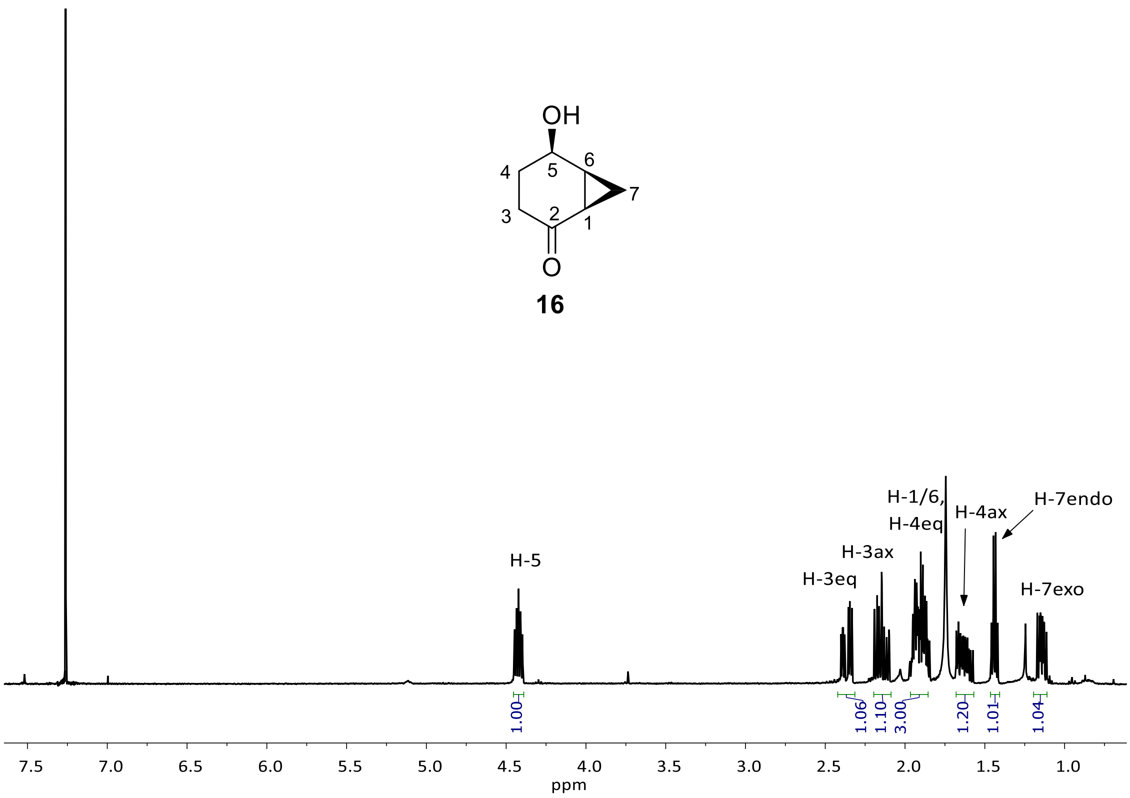

^{1}H NMR (400 MHz, $CDCl_3$)

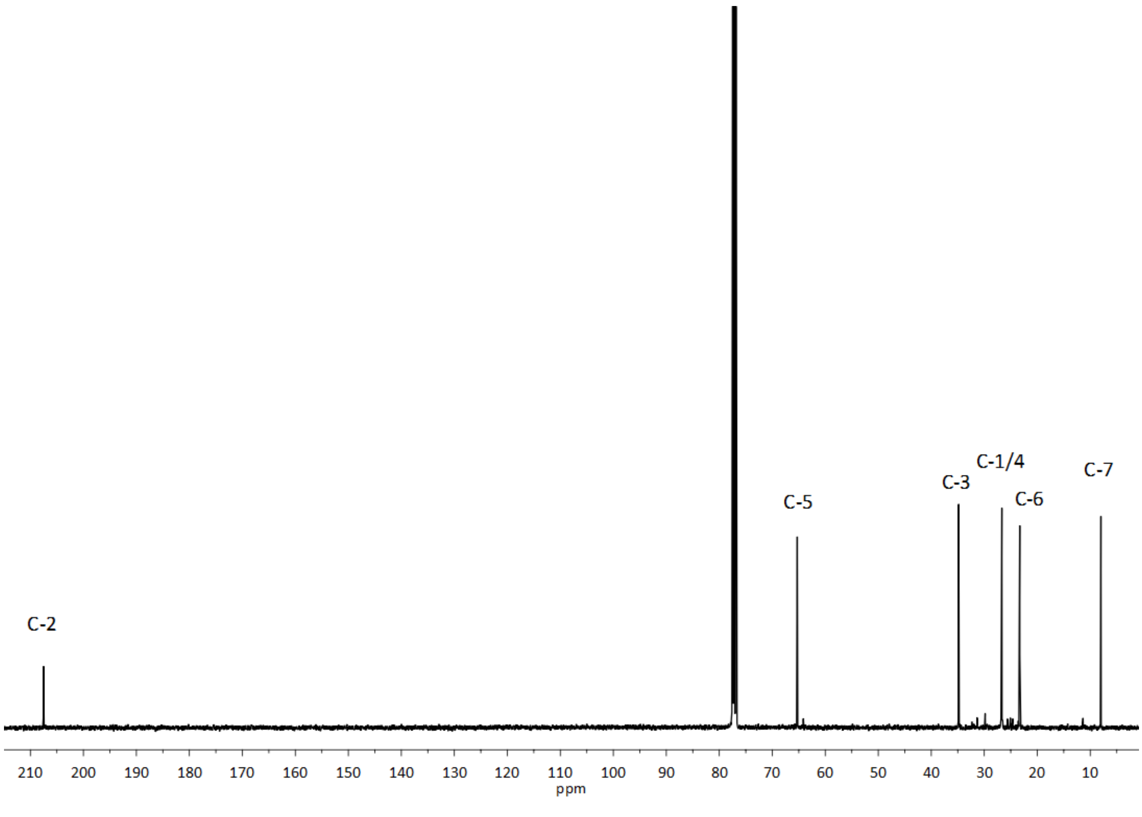

^{13}C NMR (100 MHz, $CDCl_3$)

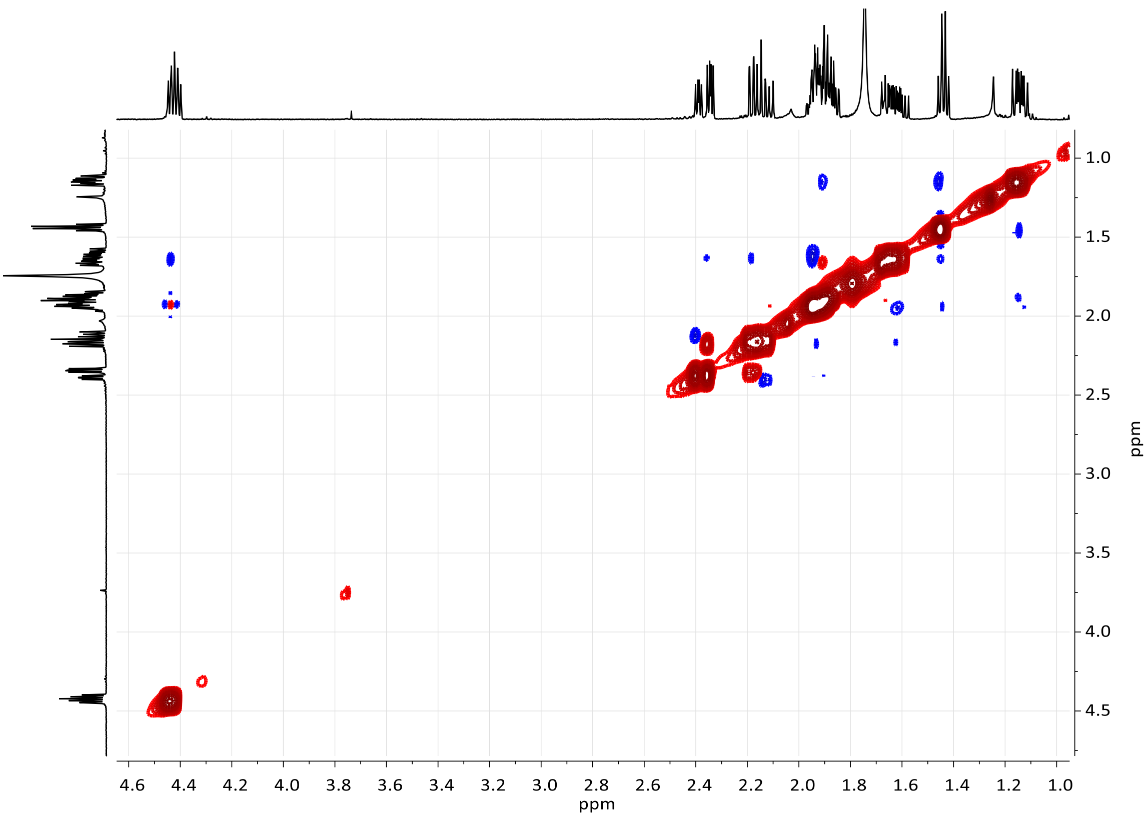

NOESY (400 MHz, $CDCl_3$)

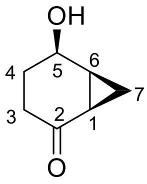

16

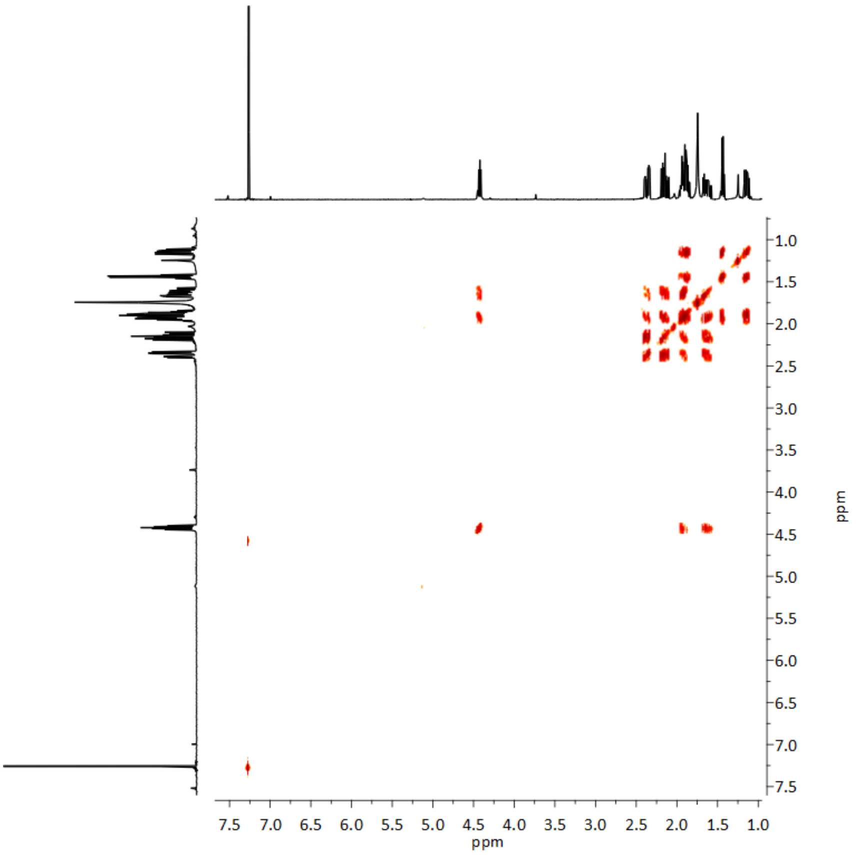

COSY (400 MHz, $CDCl_3$)

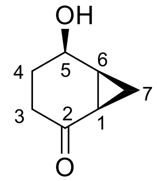

16

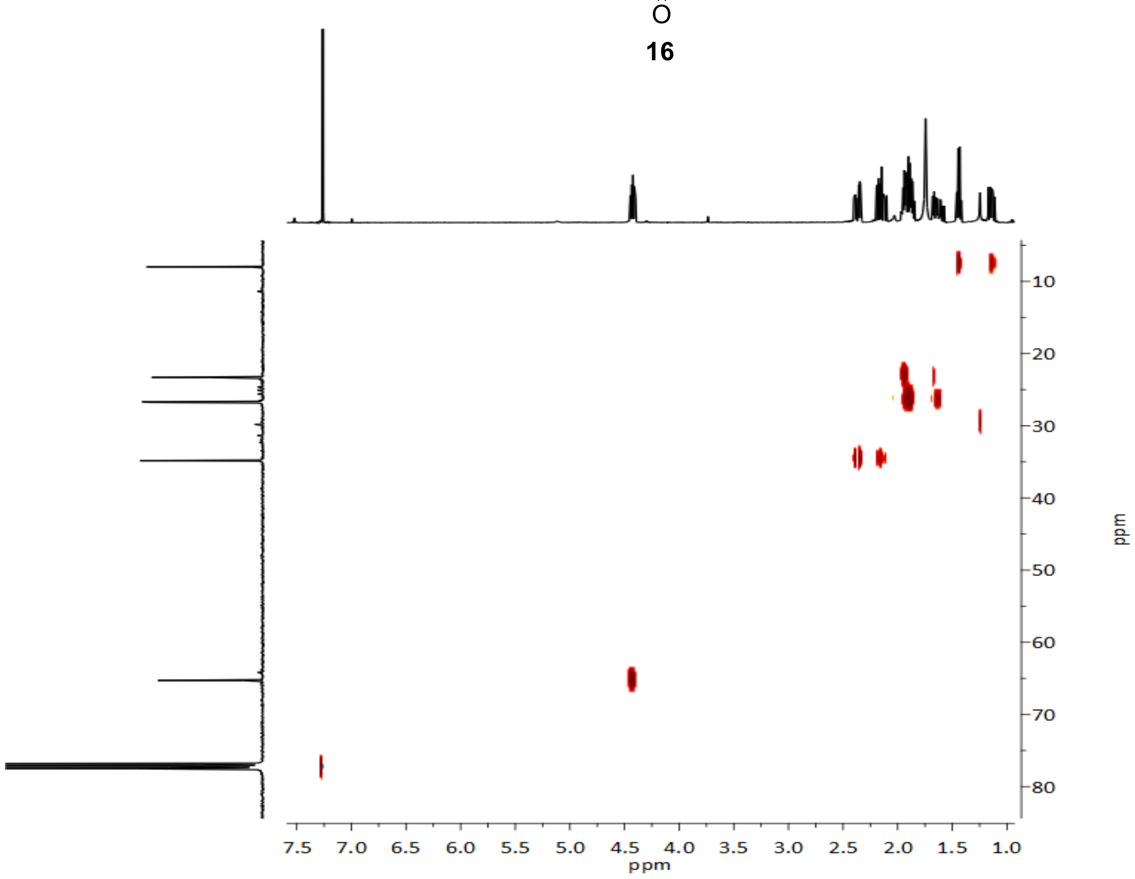

HSQC (400 MHz, $CDCl_3$)

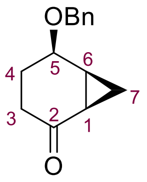

18

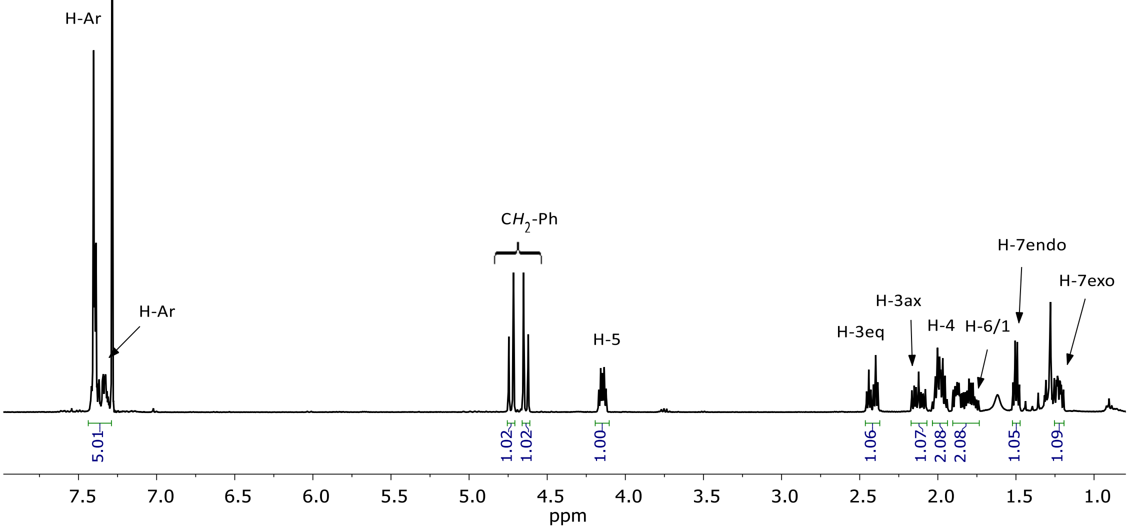

^{1}H NMR (400 MHz, $CDCl_3$)

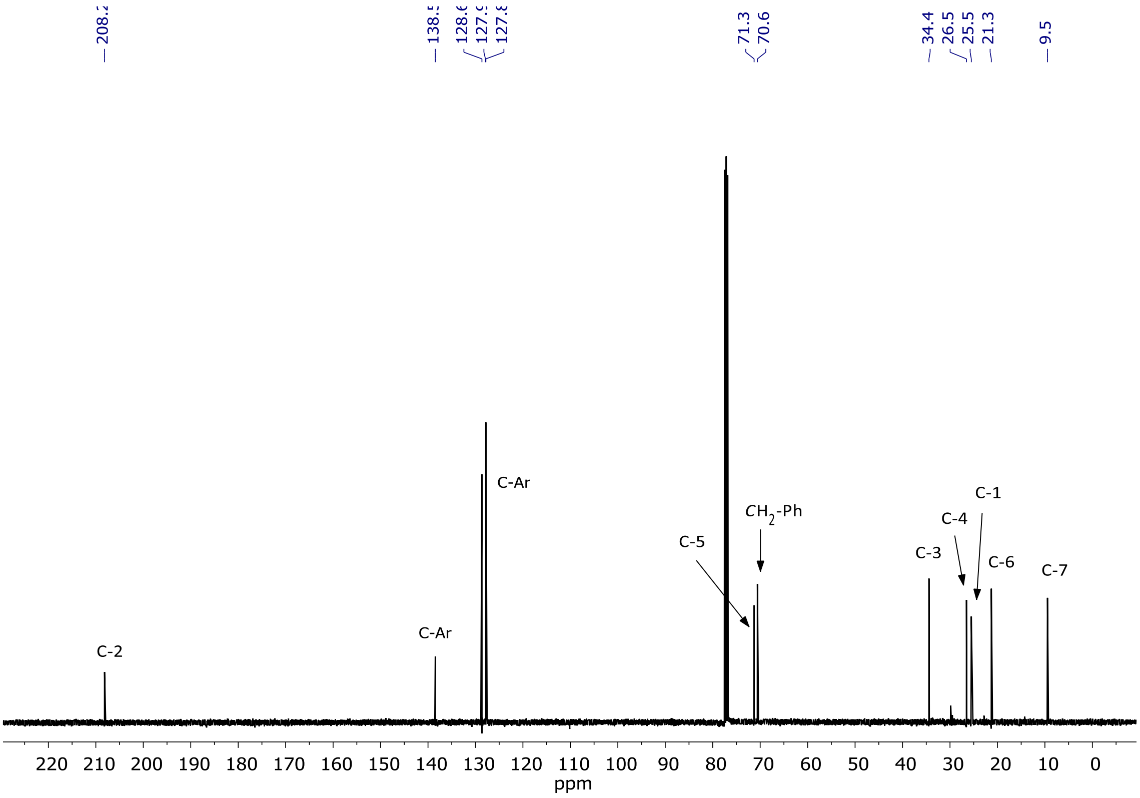

^{13}C NMR (100 MHz, $CDCl_3$)

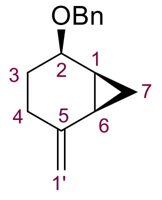

19

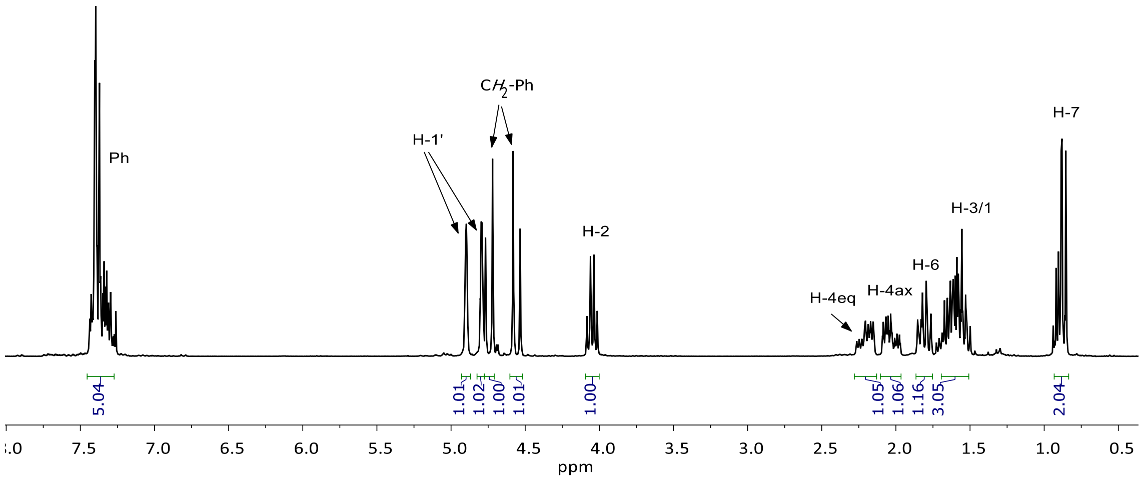

^{1}**H NMR** (400 MHz, $CDCl_3$)

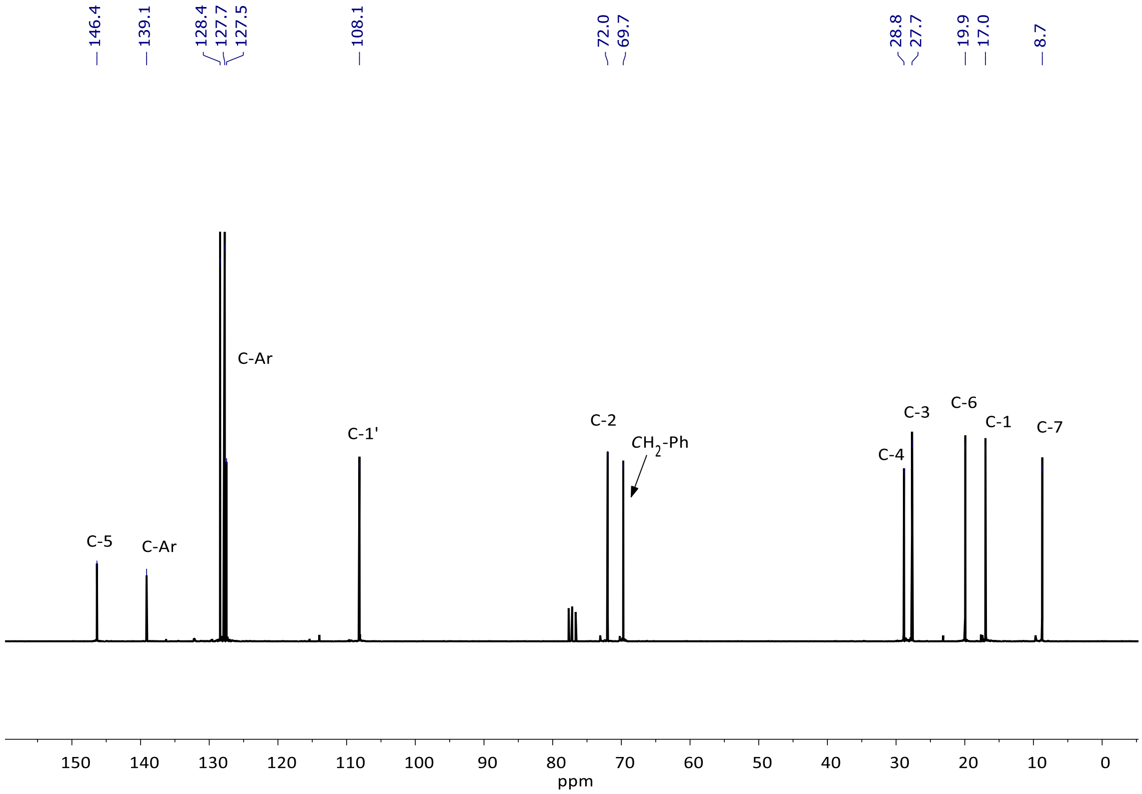

^{13}**C NMR** (100 MHz, $CDCl_3$)

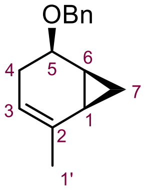

20

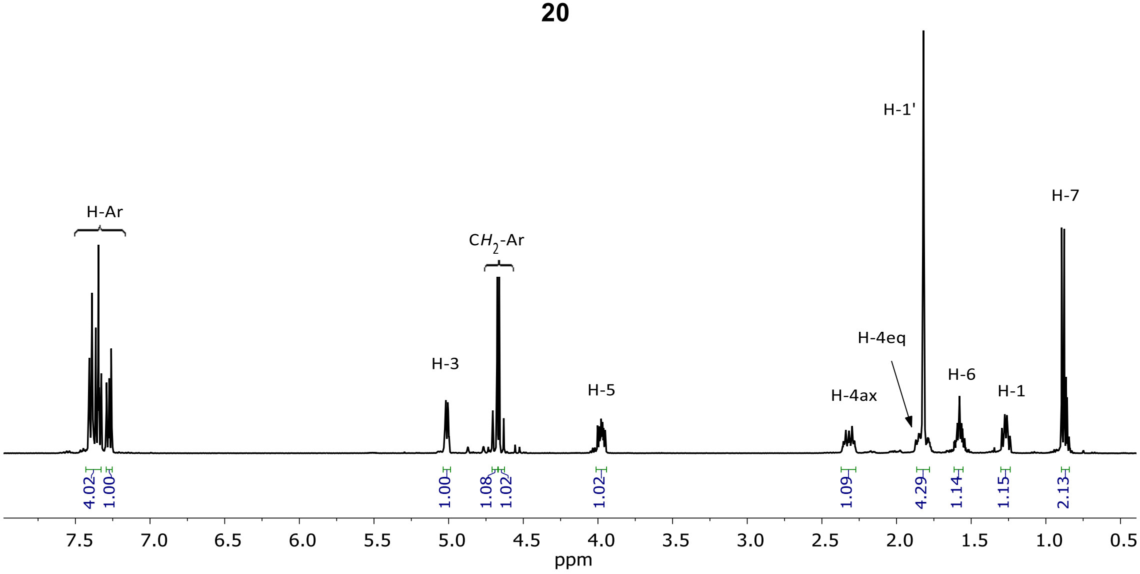

^{1}H-NMR (400 MHz, $CDCl_3$)

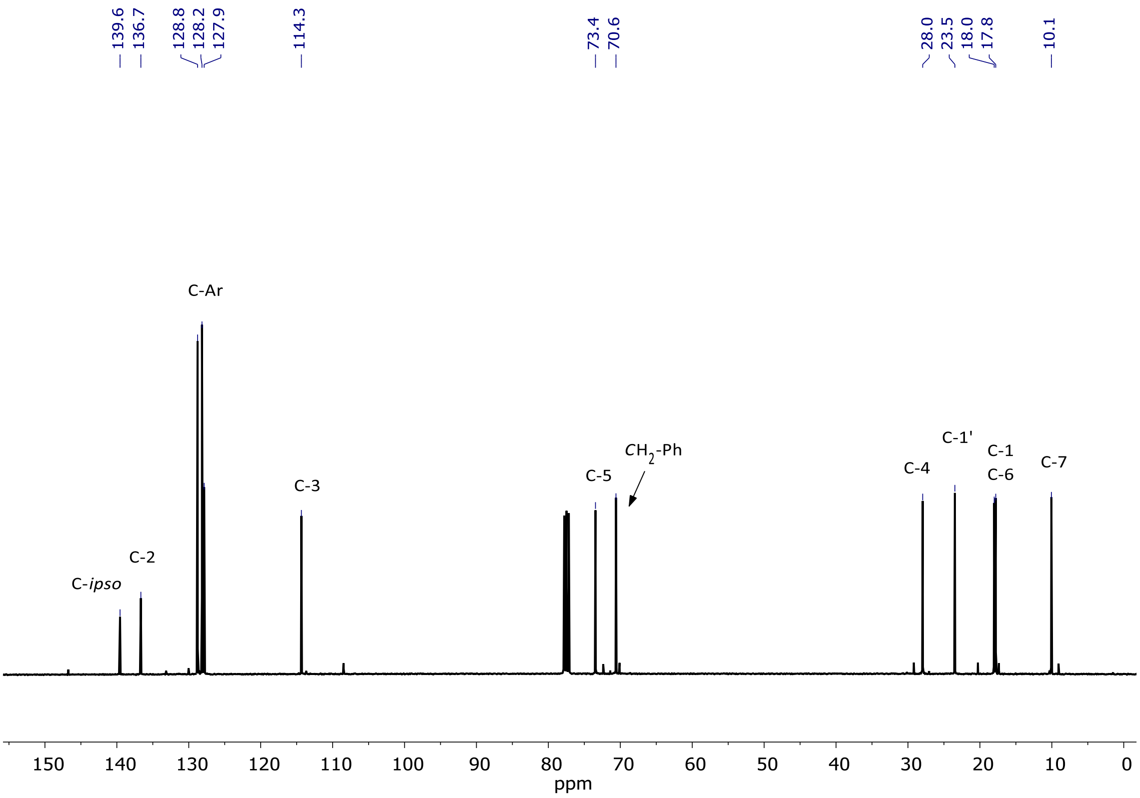

^{13}C NMR (100 MHz, $CDCl_3$)

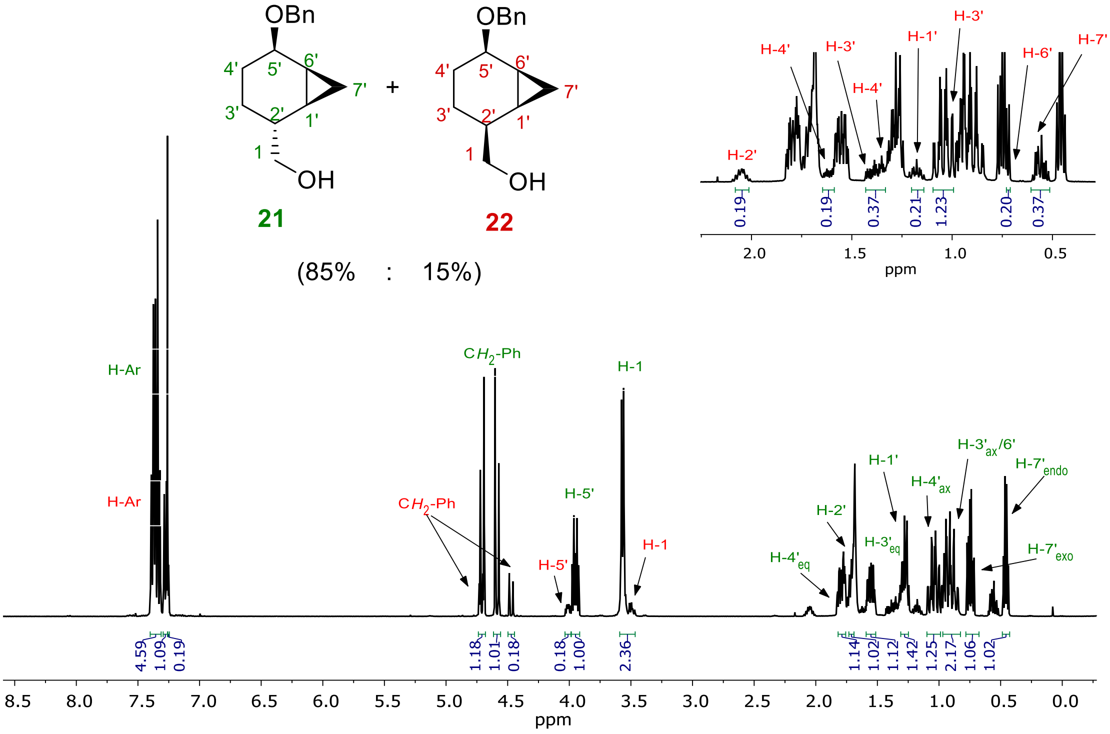

^{1}H NMR (400 MHz, $CDCl_3$)

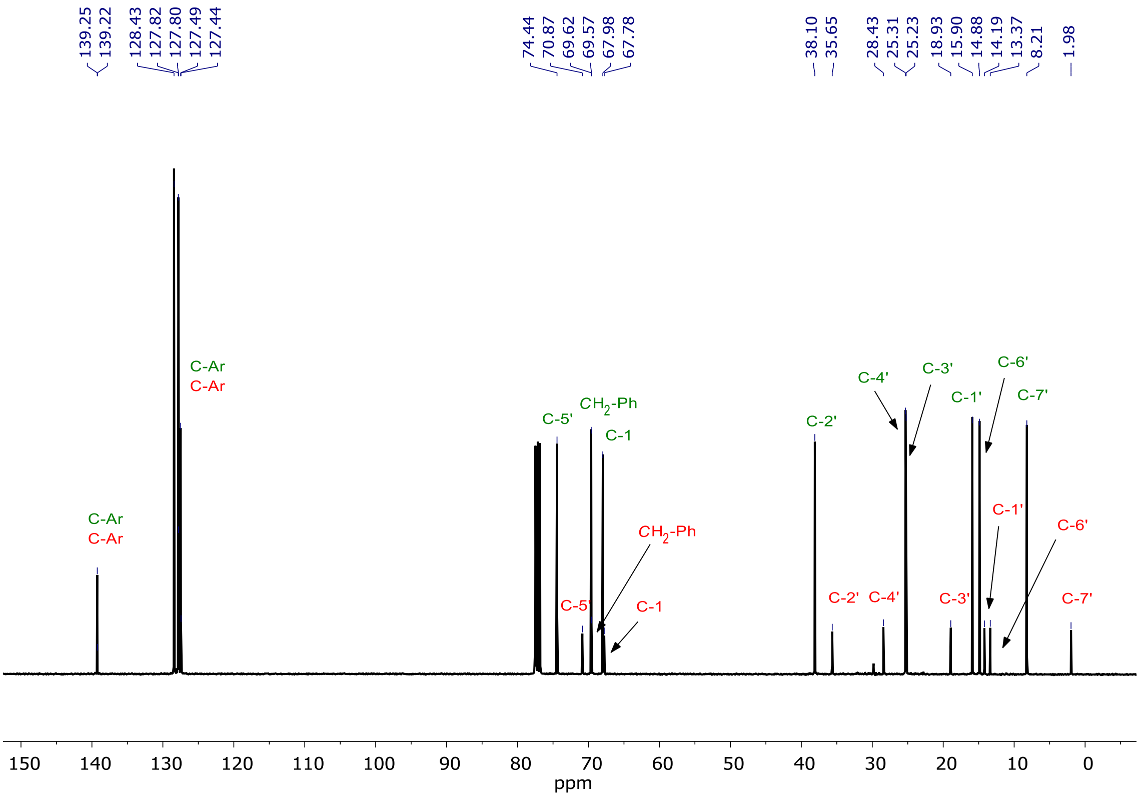

^{13}C NMR (100 MHz, $CDCl_3$)

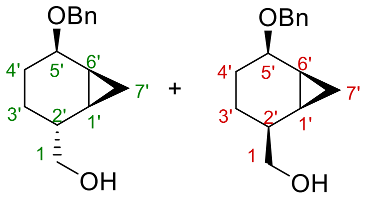

21 **22**

(85% : 15%)

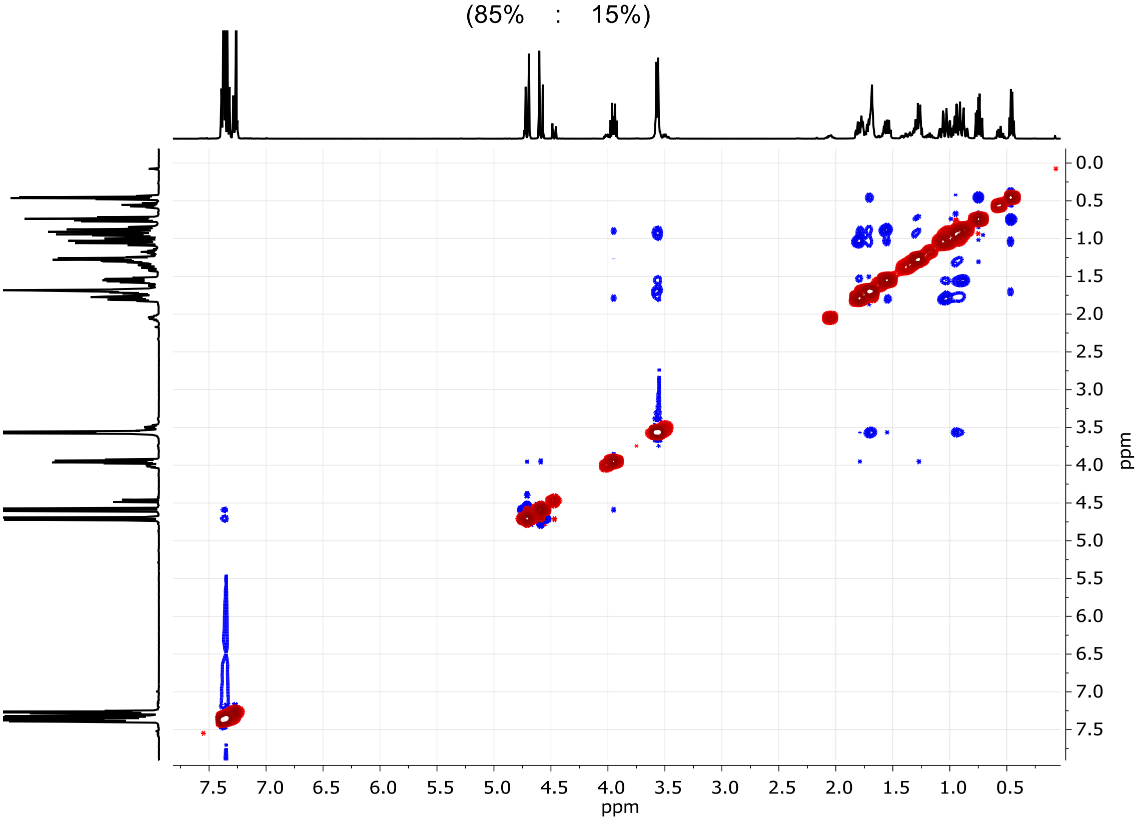

NOESY (400 MHz, $CDCl_3$)

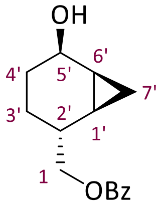

23

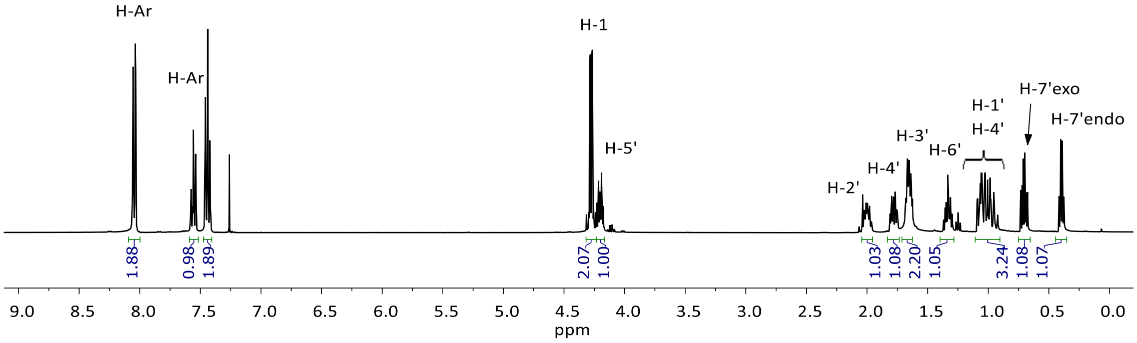

^{1}H NMR (400 MHz, $CDCl_3$)

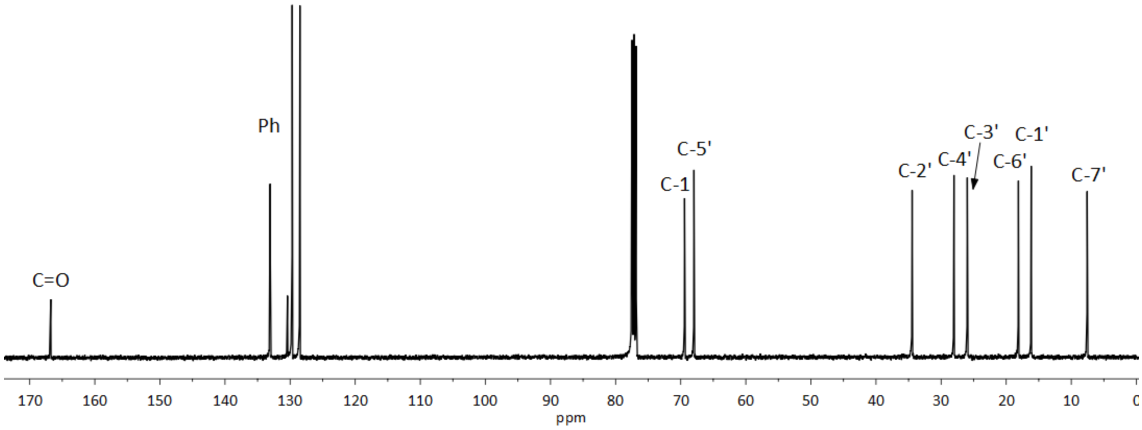

^{13}CNMR (100 MHz, $CDCl_3$)

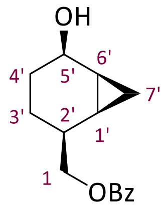

24

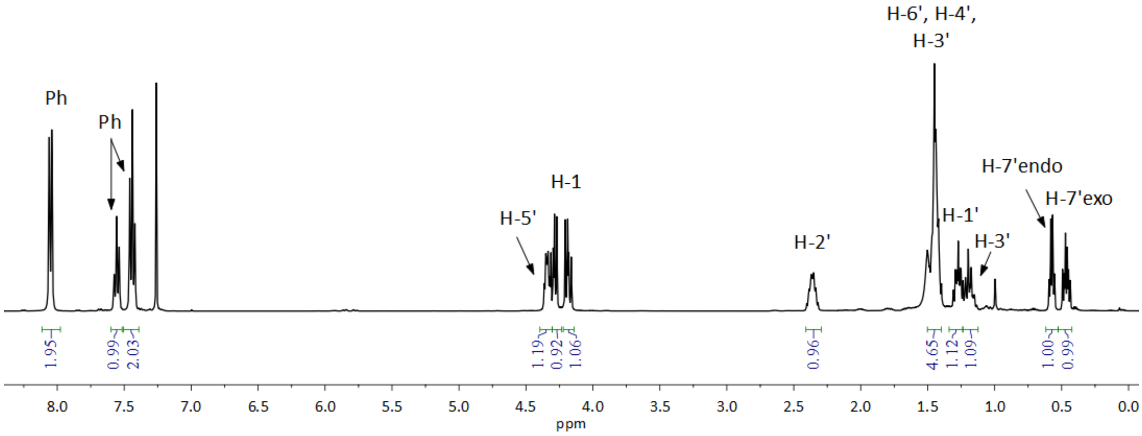

^{1}H NMR (400 MHz, $CDCl_3$)

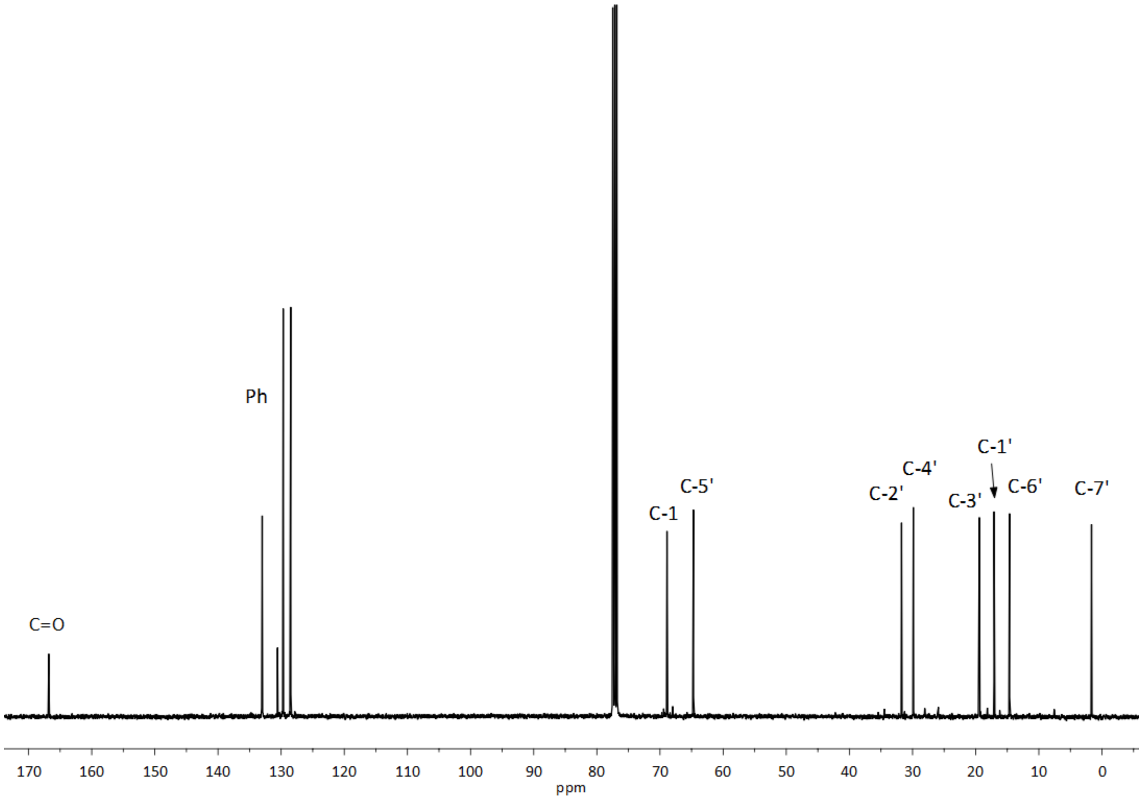

^{13}C NMR (100 MHz, $CDCl_3$)

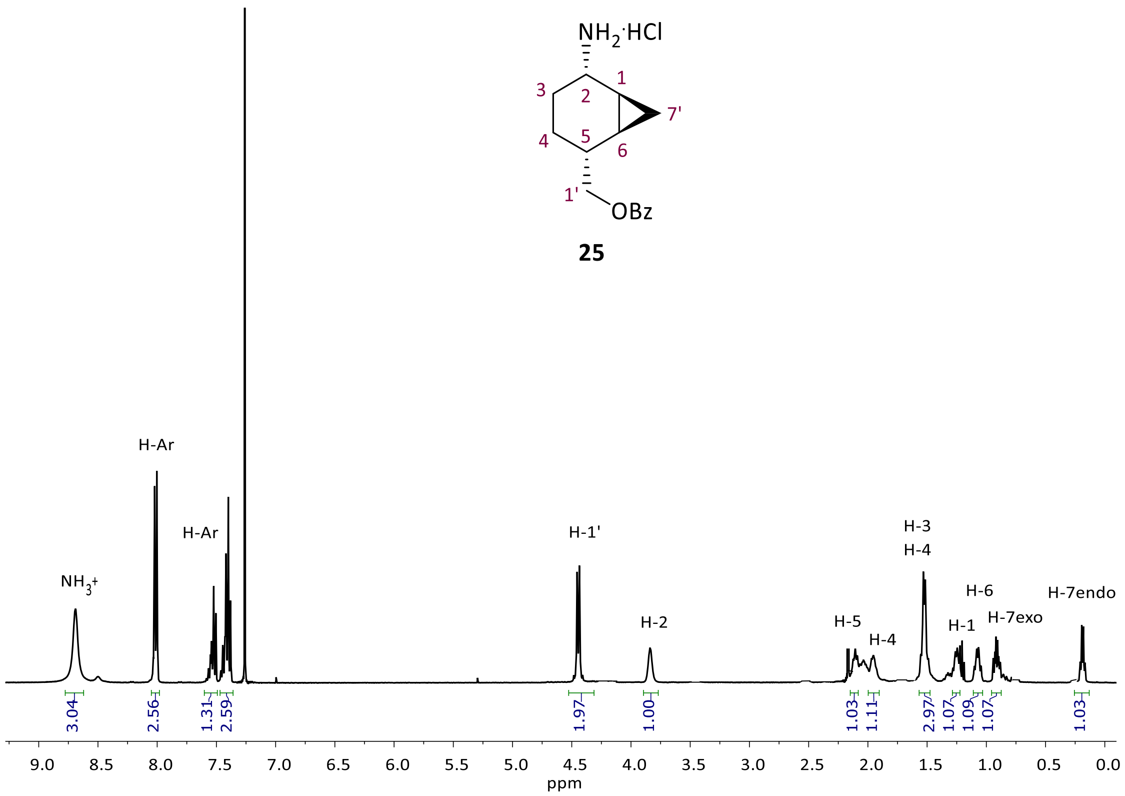

^{1}H NMR (400 MHz, $CDCl_3$)

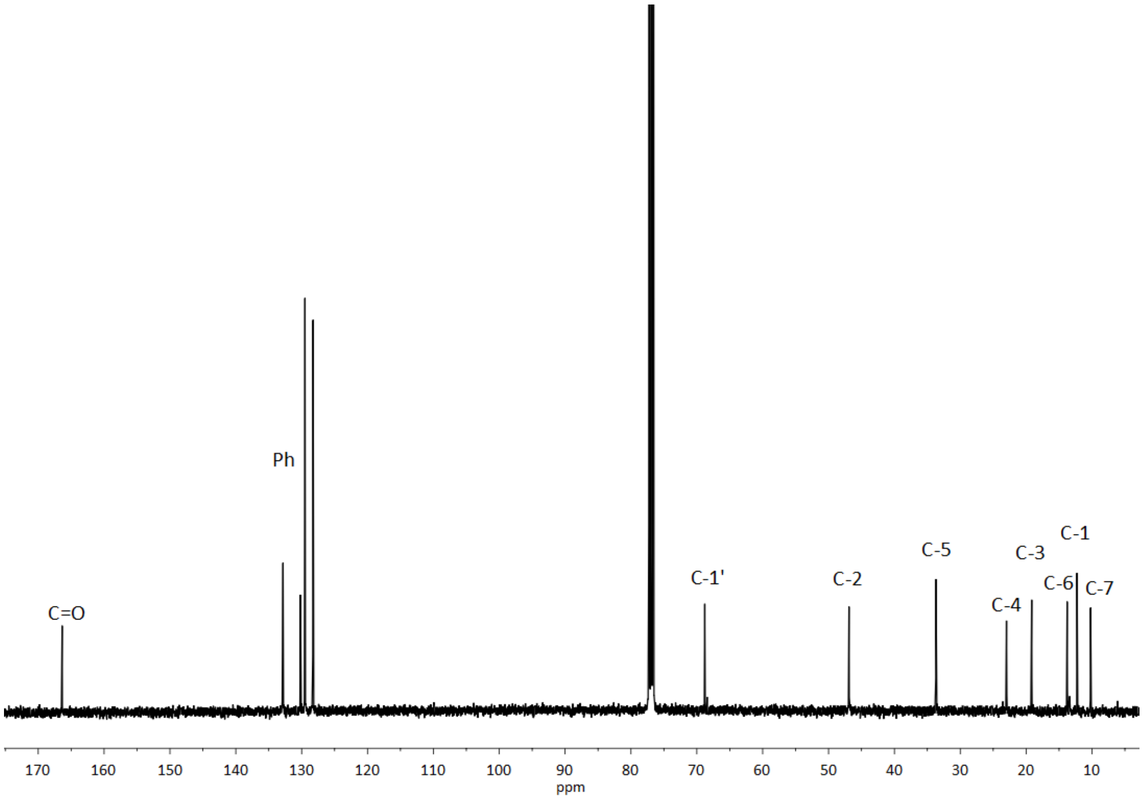

^{13}C NMR (100 MHz, $CDCl_3$)

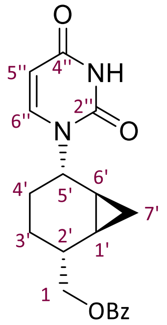

27

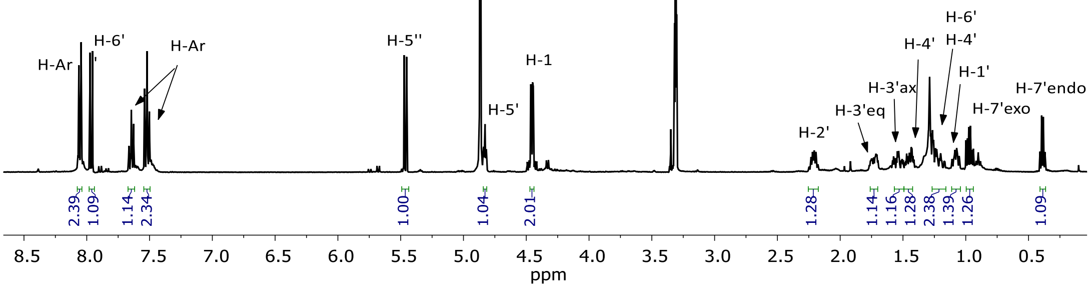

^{1}H NMR (400 MHz, MeOH-d_4)

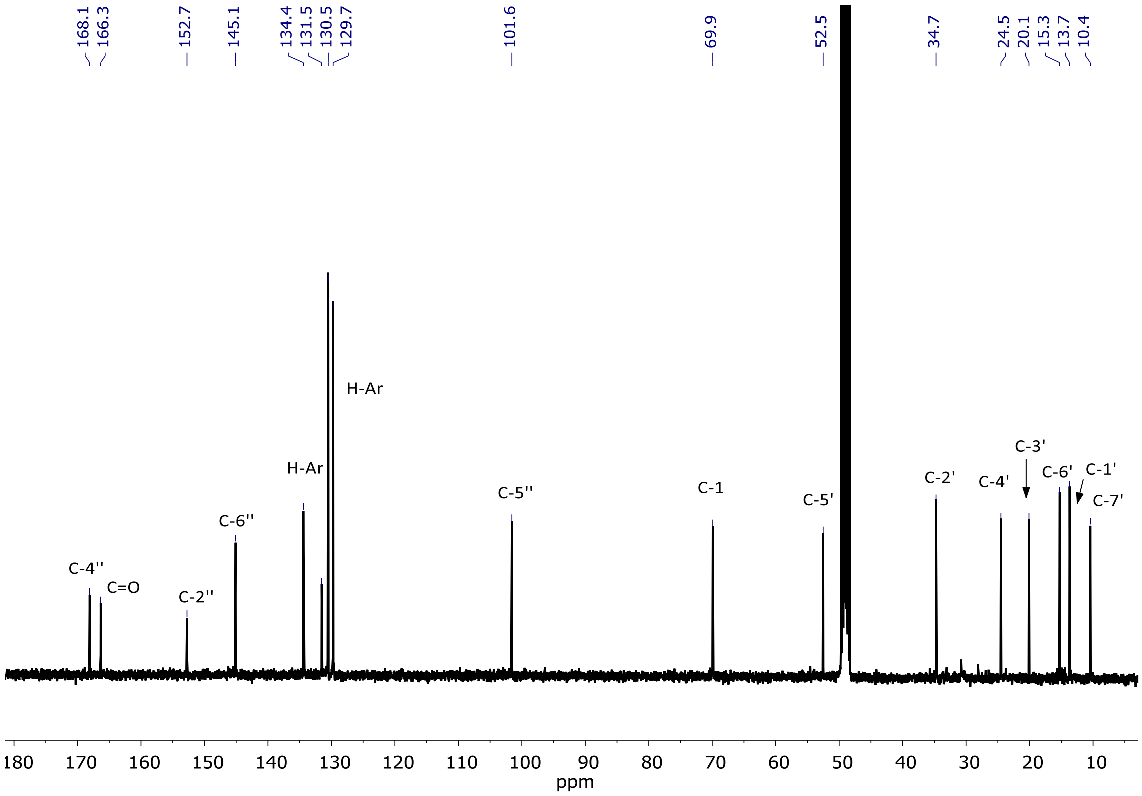

^{13}C NMR (100 MHz, MeOH-d_4)

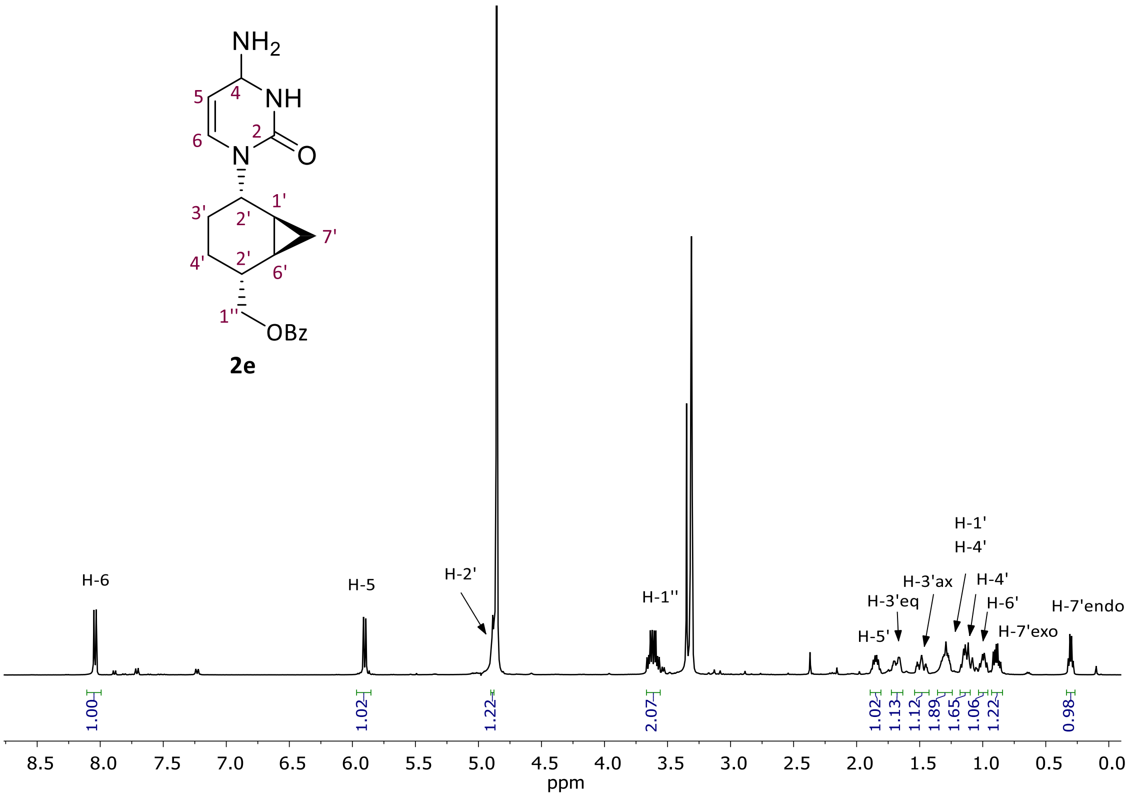

^{1}H NMR (400 MHz, MeOH-d_4)

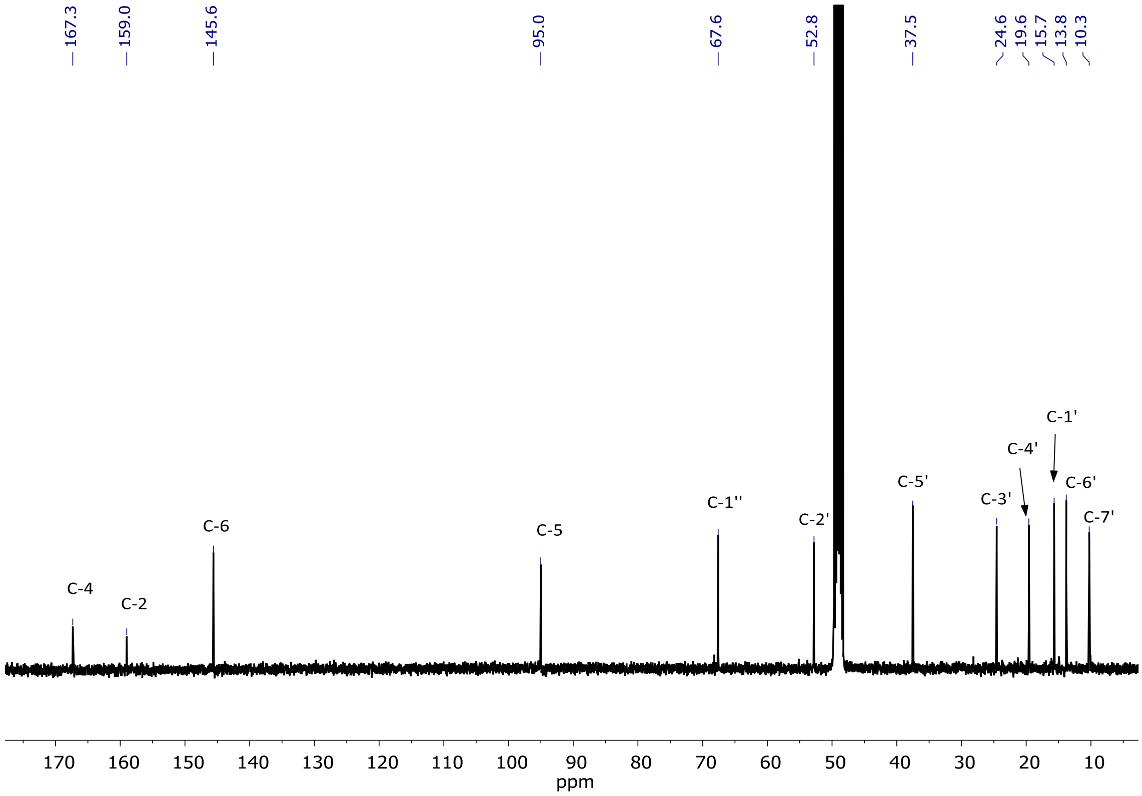

^{13}C NMR (100 MHz, MeOH-d_4)

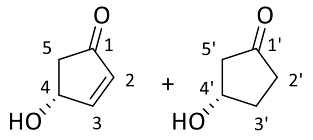

(*R*)-**S5** **S6**

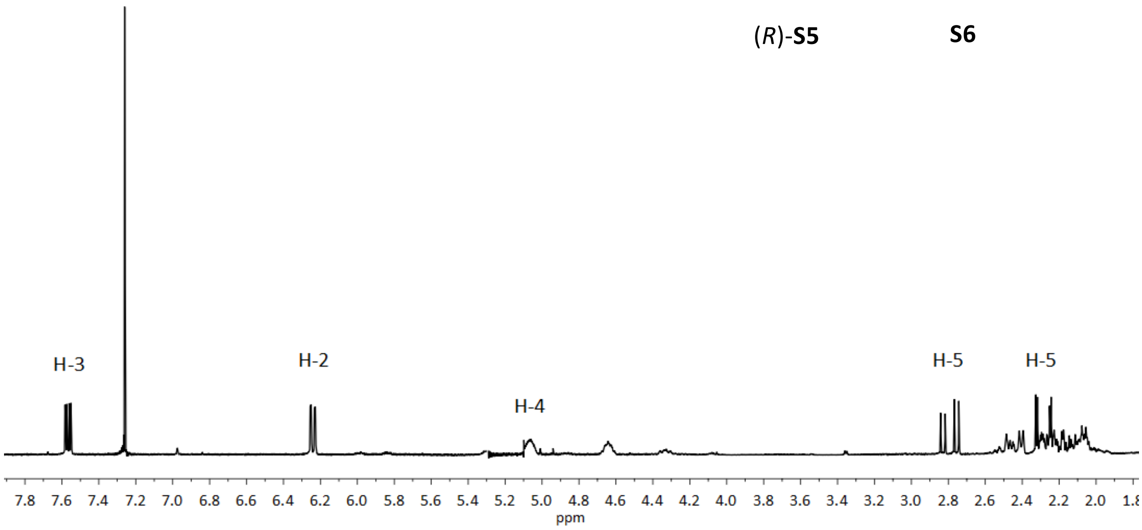

^{1}**H NMR** (250 MHz, $CDCl_3$)

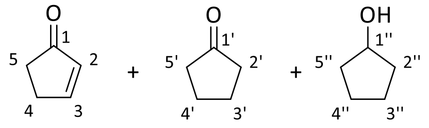

S2 **S7** **S8**

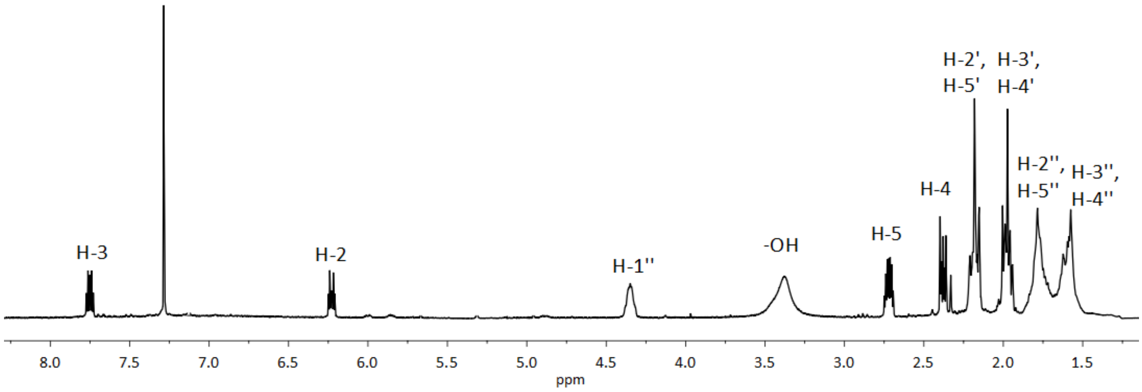

^{1}**H NMR** (250 MHz, $CDCl_3$)

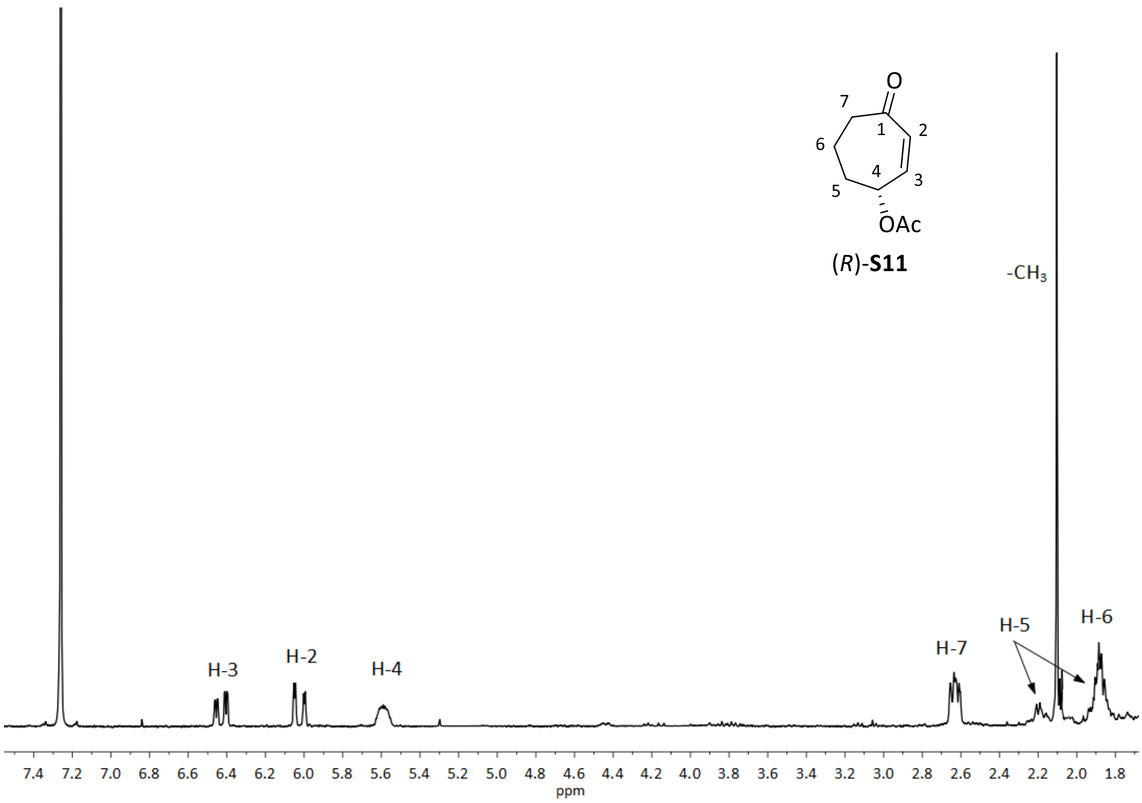

(*R*)-**S11**

^{1}**H NMR** (250 MHz, $CDCl_3$)

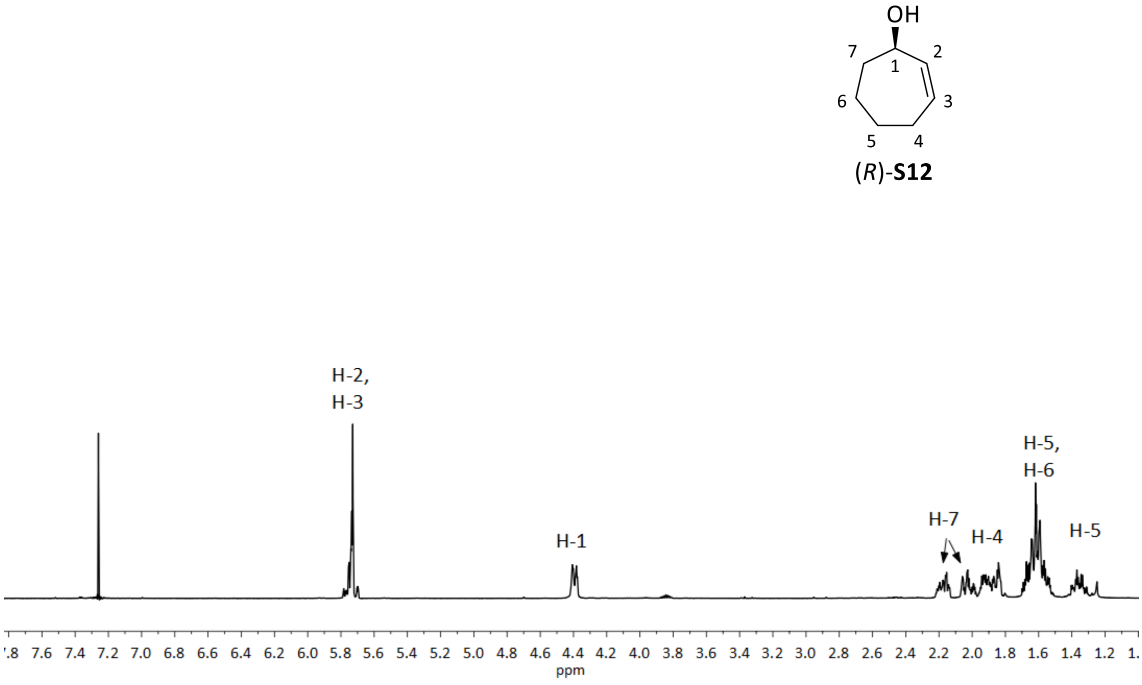

(*R*)-**S12**

^{1}**H NMR** (400 MHz, $CDCl_3$)

Enantiomeric purity

The enantiomeric excess (ee) was determined by chiral high-pressure liquid chromatography (CHPLC), using a chiral column Daicel IC[i] (0.46 cm x 0.25 cm) and a mixture of hexane:isopropanol 97:3 as eluent in 1ml/min flow rate

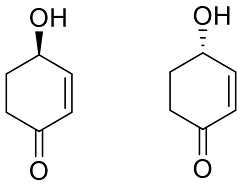

(*R*)-**1a** (*S*)-**1a**

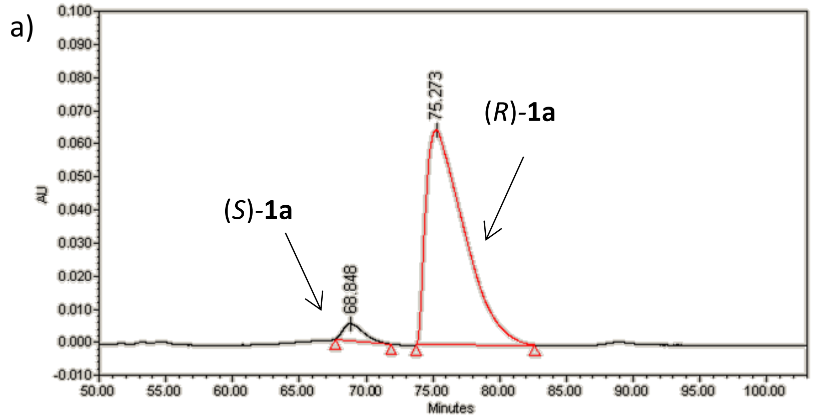

Compound	Retention time (min)	Area (µV/sec)	Area (%)
**(*S*)-1a**	68.848	565378	4.14
**(*R*)-1a**	75.273	13074831	95.86

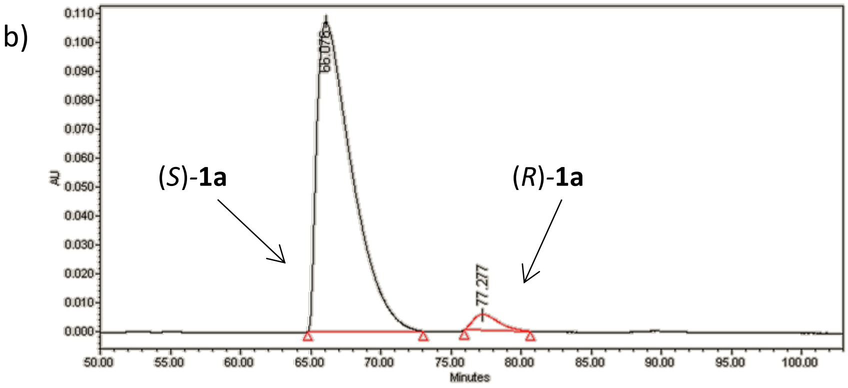

Compound	Retention time (min)	Area (µV/sec)	Area (%)
**(*S*)-29**	66.076	18846502	96.22
**(*R*)-29**	77.277	739663	3.78

Figure S1. Chiral high-pressure liquid chromatography (CHPLC) of a) (*R*)-**1a** and b) (*S*)-**1a**.

[i] Chiral column Daicel IC is based on cellulose tris(3,5-dichlorophenyl)carbamate, which is immobilized on to a wide pore silica matrix.

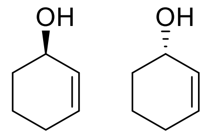

(*R*)-**11** (*S*)-**11**

Column: Daicel IC (0.46 cm x 0.25 cm); eluent: hexane-isopropanol (97:3); flow rate: 0.5 mL/min.

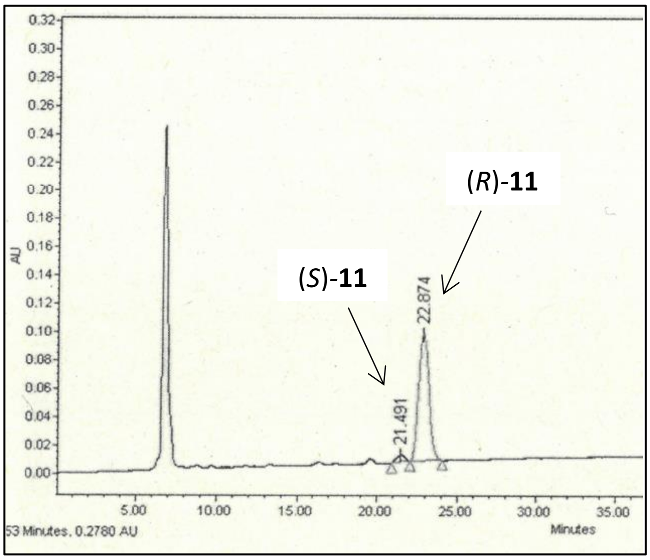

Compound	Retention time (min)	Area (%)
**(*S*)-11**	21.491	4.02
**(*R*)-11**	22.874	95.98

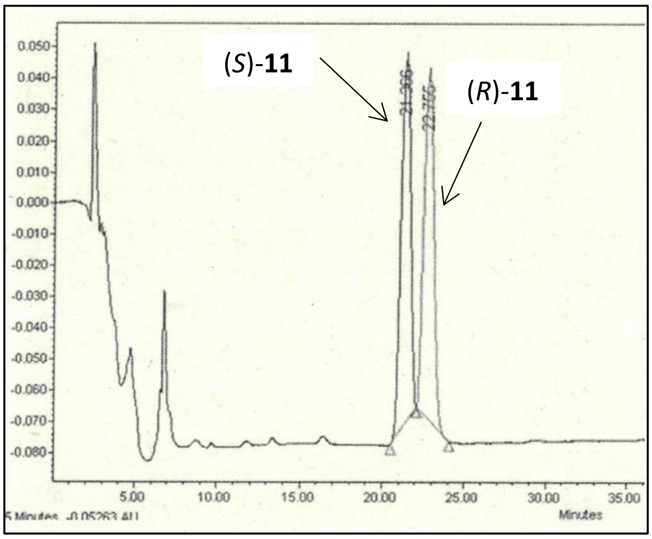

Compound	Retention time (min)	Area (%)
**(*S*)-11**	21.336	50.15
**(*R*)-11**	22.755	49.85

Figure S2. Chiral high-pressure liquid chromatography (CHPLC) of a) (*R*)-**11** and b) *rac*-**11**.

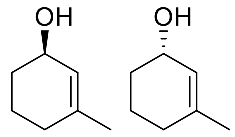

(*R*)-**13** (*S*)-**13**

Column: Daicel IC (0.46 cm x 0.25 cm); eluent: hexane-isopropanol (98.5:1.5); flow rate: 0.4 mL/min.

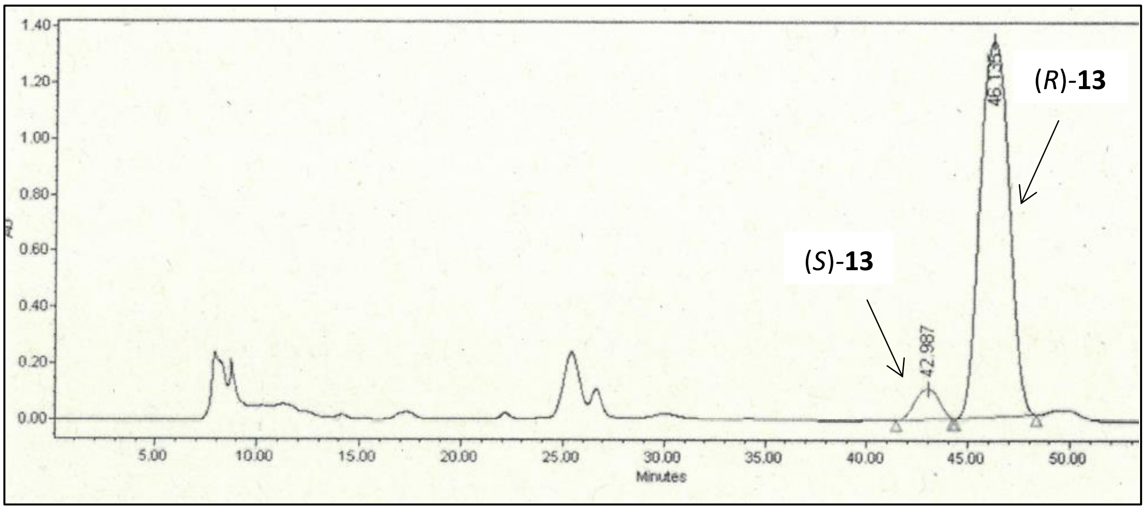

Compound	Retention time (min)	Area (%)
**(*S*)-13**	42.987	6.17
**(*R*)-13**	46.135	93.83

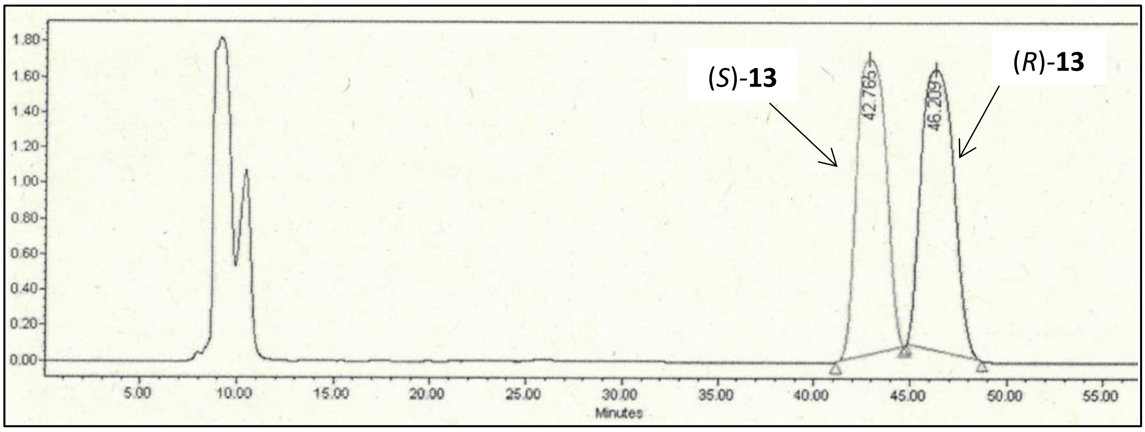

Compound	Retention time (min)	Area (%)
**(*S*)-13**	42.765	48.89
**(*R*)-13**	46.209	51.11

Figure S3. Chiral high-pressure liquid chromatography (CHPLC) of a) (*R*)-**13** and b) *rac*-**13**.

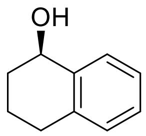

(*R*)-**15**

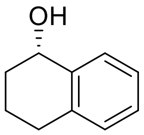

(*S*)-**15**

Column: Daicel Chiralpack OD-H[ii] (0.46 cm x 0.25 cm); eluent: hexane-isopropanol (96:4); flow rate: 0.5 mL/min.

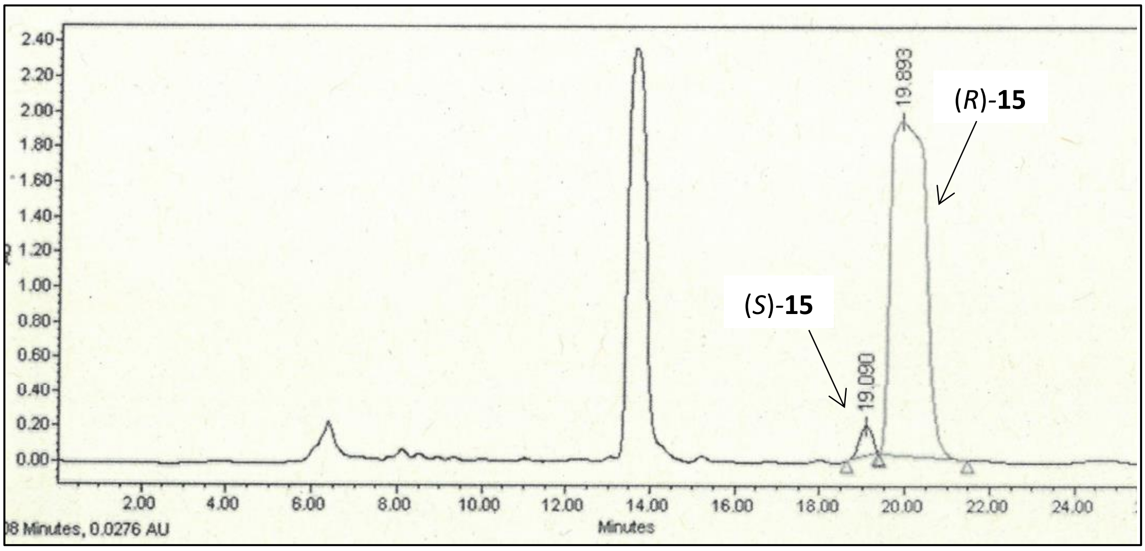

Compound	Retention time (min)	Area (%)
**(*S*)-15**	19.090	3.17
**(*R*)-15**	19.893	96.83

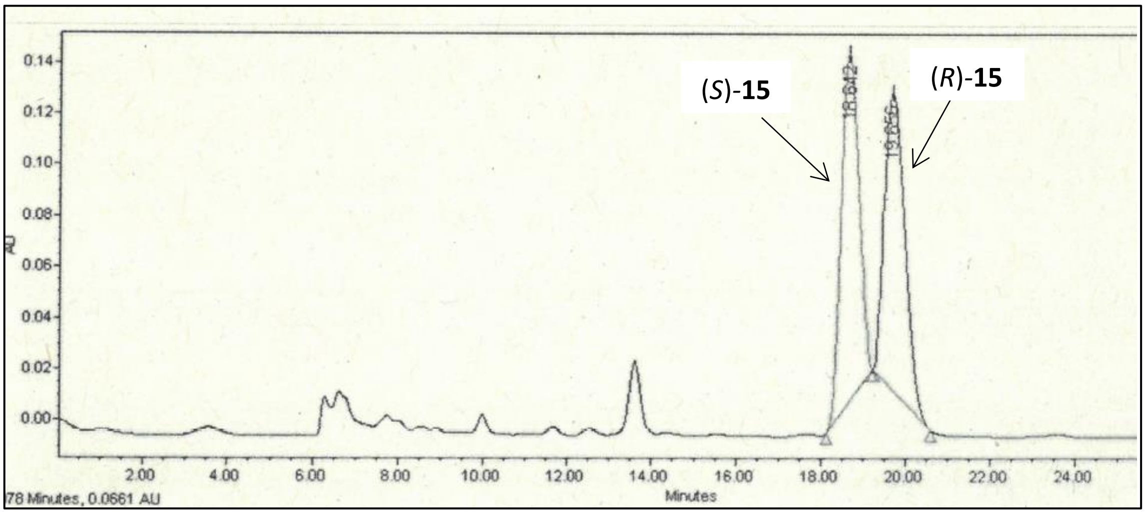

Compound	Retention time (min)	Area (%)
**(*S*)-15**	18.642	50.26
**(*R*)-15**	19.656	49.71

Figure S4. Chiral high-pressure liquid chromatography (CHPLC) of a) (*R*)-**15** and b) *rac*-**15**.

[ii] Chiral column Daicel OD-H is based on cellulose tris(3,5-dimethylphenyl)carbamate, which is coated on 5 µm silica-gel.

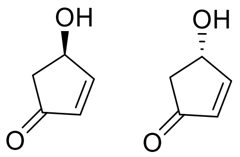

(R)-**S5** (S)-**S5**

Column: Daicel IC (0.46 cm x 0.25 cm); eluent: hexane-isopropanol (97:3); flow rate: 1.0 mL/min.

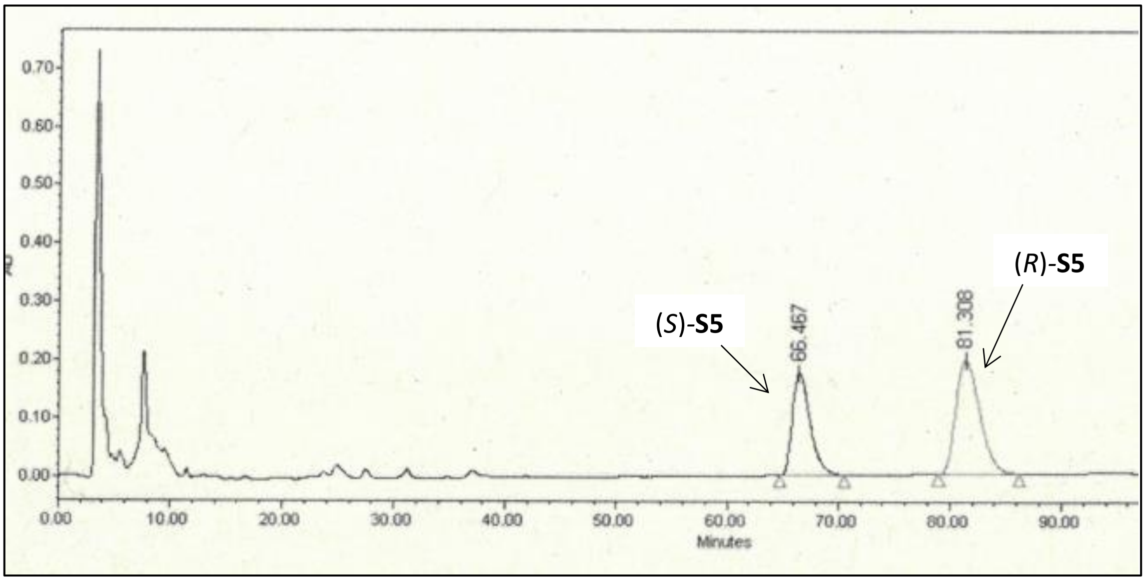

Compound	Retention time (min)	Area (%)
**(*S*)-S5**	66.467	39.94
**(*R*)-S5**	81.308	60.06

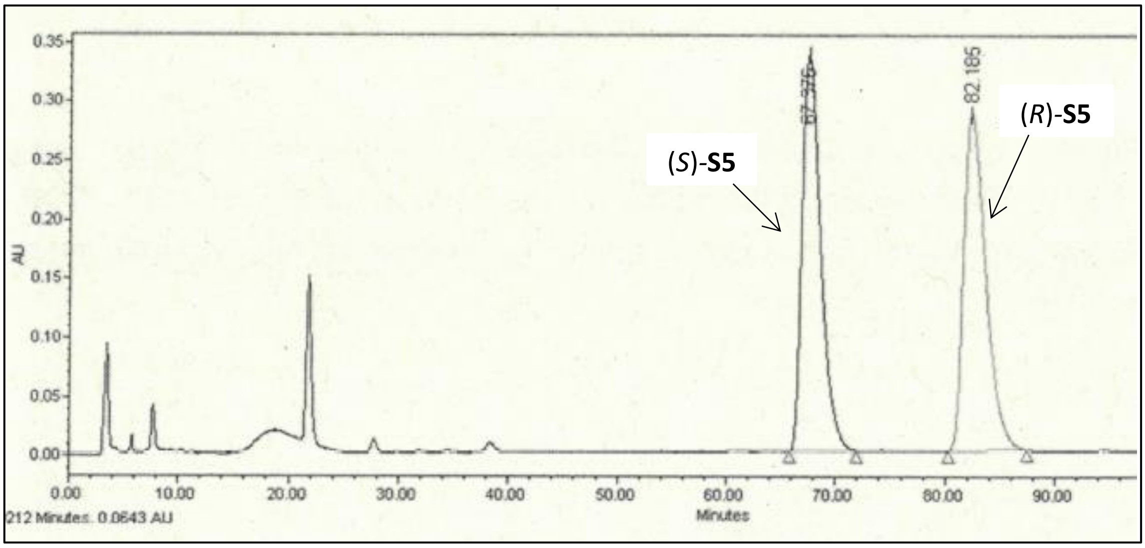

Compound	Retention time (min)	Area (%)
**(*S*)-S5**	67.376	49.96
**(*R*)-S5**	82.185	50.04

Figure S5. Chiral high-pressure liquid chromatography (CHPLC) of a) (R)-**S5** and b) *rac*-**S5**.

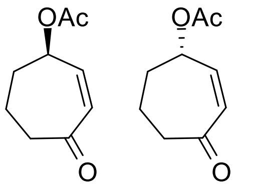

(*R*)-**S11** (*S*)-**S11**

Column: Daicel IC (0.46 cm x 0.25 cm); eluent: hexane-isopropanol (95.8:4.2); flow rate: 1.0 mL/min.

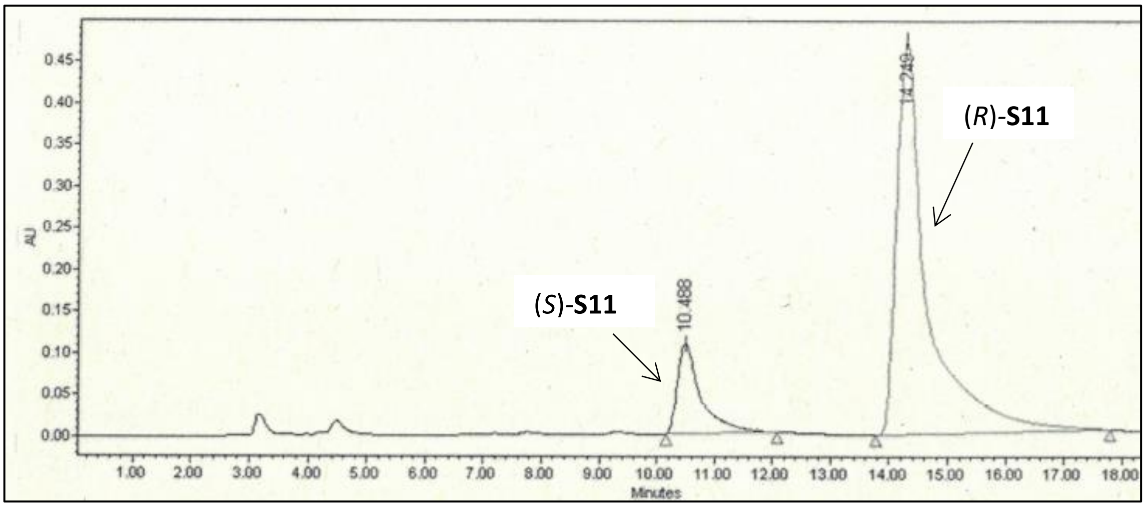

Compound	Retention time (min)	Area (%)
**(*S*)-S11**	10.488	14.91
**(*R*)-S11**	14.249	85.09

Figure S6. Chiral high-pressure liquid chromatography (CHPLC) of a) (*R*)-**S9**.

Chiral high-pressure liquid chromatography (CHPLC) of *rac*-**S11** Ref. [1].

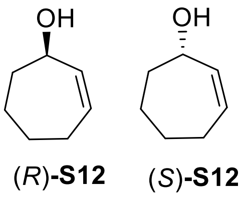

Column: Daicel IC (0.46 cm x 0.25 cm); eluent: hexane-isopropanol (98.5:1.5); flow rate: 0.4 mL/min.

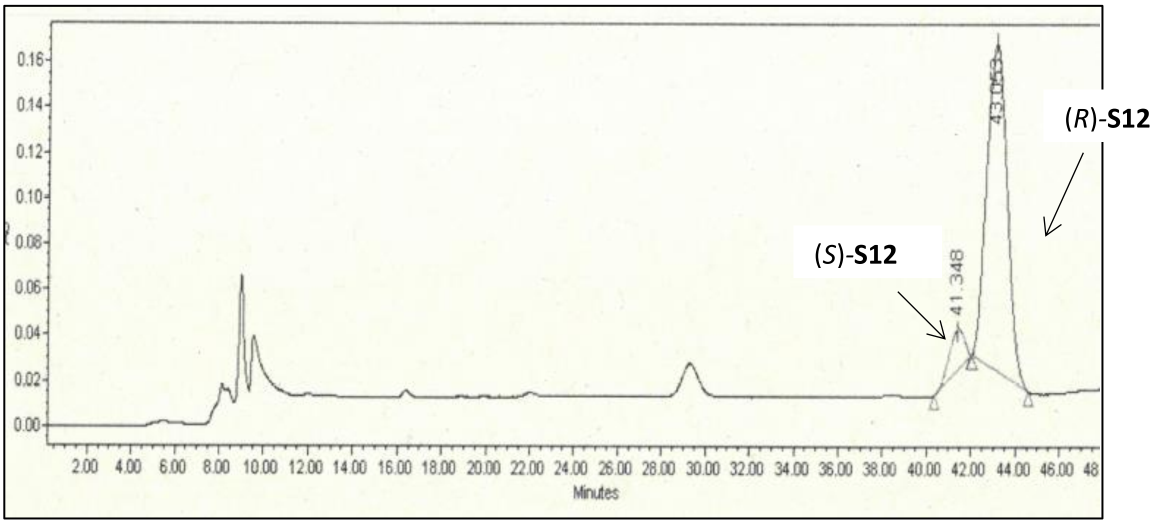

Compound	Retention time (min)	Area (%)
**(*S*)-S12**	41.348	8.41
**(*R*)-S12**	43.053	91.59

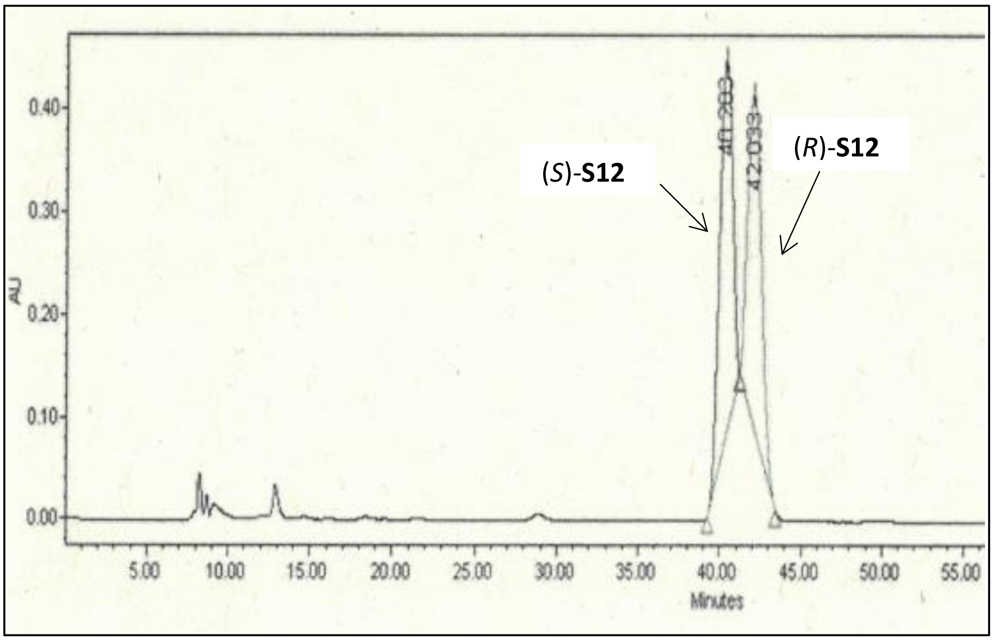

Compound	Retention time (min)	Area (%)
**(*S*)-S12**	40.283	50.77
**(*R*)-S12**	42.033	49.23

Figure S7. Chiral high-pressure liquid chromatography (CHPLC) of a) (*R*)-**S12** and b) *rac*-**S12**.

Antiviral Activity Assays

Methods

Compound **2e** was evaluated for its inhibitory activity against herpes simplex virus type 1 (HSV-1) strain KOS and herpes simplex virus type 2 (HSV-2) strain G. The antiviral assays were based on inhibition of virus-induced cytopathicity in human embryonic lung (HEL) fibroblasts. Adherent human embryonic lung fibroblast cell cultures (HEL-299) were used (ATCC CCL 137). These cells were obtained from a male in 1965. The cells were used for research purposes only, and cultivated in a containment level 2 in accordance to the Advisory Committee on Dangerous Pathogens (ACDP) Guidelines.[3]

Confluent cell cultures in microtitre 96-well plates were inoculated with 100 CCID50 of the virus (1 CCID50 being the dose of the virus sufficient to infect 50% of the cell cultures) in the presence of varying concentrations of the test compounds. Viral cytopathicity was recorded as soon as it reached completion in the control virus-infected cell cultures that had not been treated with the test compounds. Antiviral activity is expressed as EC50, i.e., the compound concentration required to suppress virus-induced cytopathogenicity by 50%.

Table S2. Cytotoxicity and anti Herpes Simplex Virus activity in HEL (human embryonic lung) cell cultures.

**Compound**	**Conc. Unit**	**Cytotoxicity**[a]	**Antiviral EC_{50}**[b]	
			Herpes simplex virus-1 (KOS)	Herpes simplex virus-2 (G)
**2e**	µM	>100	>100	>100
Brivudin	µM	>250	0.04	112
Cidofovir	µM	>250	2.0	1.4
Acyclovir	µM	>250	0.4	0.4
Ganciclovir	µM	>100	0.02	0.05

[a] Minimum cytotoxic concentration required to cause a microscopically detectable alteration of normal cell morphology.[b] Required to reduce virus-induced cytopathogenic effect by 50%.

References

1. Kawasumi, M.; Kanoh, N.; Iwabuchi, Y. Concise Entry to Both Enantiomers of 8-Oxabicyclo[3.2.1]oct-3-en-2-one Based on Novel Oxidative Etherification: Formal Synthesis of (+)-Sundiversifolide. *Org. Lett.* **2011**, *13*. 3620-3623.

2. Gais, H. J.; Jagusch, T.; Spalthoff, N.; Gerhards, F.; Frank, M.; Raabe, G. Highly Selective Palladium Catalyzed Kinetic Resolution and Enantioselective Substitution of Racemic Allylic Carbonates with Sulfur Nucleophiles: Asymmetric Synthesis of Allylic Sulfides, Allylic Sulfones, and Allylic Alcohols. *Chem. Eur. J.* **2003**, *9*, 4202–4221.

3. Geraghty, R. J.; Capes-Davis, A.; Davis, J. M.; Downward, J. Freshney, R. I.; Knezevic, I.; Lovell-Badge, R.; Masters, J. R. W.; Meredith, J.; Stacey, G. N.; Thraves, P.; Vias, M. Guidelines for the Use of Cell Lines in Biomedical Research. *Br. J. Cancer.* **2014**, 111, 1021–1046.
